# Experience with a hybrid recruitment approach of patient-facing web portal screening and subsequent phone and medical record review for a neurosurgical intervention trial for chronic ischemic stroke disability (PISCES III)

**DOI:** 10.1186/s13063-024-07988-z

**Published:** 2024-02-28

**Authors:** Brad J. Kolls, Keith W. Muir, Sean I. Savitz, Lawrence R. Wechsler, Julie G. Pilitsis, Scott Rahimi, Richard L. Beckman, Vincent Holmes, Peng R. Chen, David S. Albers, Daniel T. Laskowitz

**Affiliations:** 1 Duke Clinical Research Institute, Duke University School of Medicine, Durham, NC USA; 2 Department of Neurology, Duke University School of Medicine, Duke Box 2900 Bryan Research Building, 311 Research Drive, Durham, NC 27710 USA; 3 School of Psychology & Neuroscience, University of Glasgow, Queen Elizabeth University Hospital, Glasgow, Scotland UK; 4 Institute for Stroke and Cerebrovascular Disease, University of Texas Health Science Center, Houston, TX USA; 5 Department of Neurology, Perelman School of Medicine, University of Pennsylvania, Philadelphia, PA USA; 6 Department of Neuroscience and Experimental Therapeutics, Albany Medical College, Albany, NY USA; 7 Department of Neurosurgery, Medical College of Georgia, Augusta, GA USA; 8 ReNeuron Limited., Pencoed, Bridgend, UK; 9 The Vivian L. Smith Department of Neurosurgery, The University of Texas Health Science Center, Houston, TX USA

**Keywords:** Internet-based trial recruitment, Trial participant screening, Invasive trial recruitment, Chronic ischemic stroke, Cell based intervention, Stem cell

## Abstract

**Background:**

Recruitment of participants is the greatest risk to completion of most clinical trials, with 20–40% of trials failing to reach the targeted enrollment. This is particularly true of trials of central nervous system (CNS) therapies such as intervention for chronic stroke. The PISCES III trial was an invasive trial of stereotactically guided intracerebral injection of CTX0E03, a fetal derived neural stem cell line, in patients with chronic disability due to ischemic stroke. We report on the experience using a novel hybrid recruitment approach of a patient-facing portal to self-identify and perform an initial screen for general trial eligibility (tier 1), followed by phone screening and medical records review (tier 2) prior to a final in-person visit to confirm eligibility and consent.

**Methods:**

Two tiers of screening were established: an initial screen of general eligibility using a patient-facing web portal (tier 1), followed by a more detailed screen that included phone survey and medical record review (tier 2). If potential participants passed the tier 2 screen, they were referred directly to visit 1 at a study site, where final in-person screening and consent were performed. Rates of screening were tracked during the period of trial recruitment and sources of referrals were noted.

**Results:**

The approach to screening and recruitment resulted in 6125 tier 1 screens, leading to 1121 referrals to tier 2. The tier 2 screening resulted in 224 medical record requests and identification of 86 qualifying participants for referral to sites. The study attained a viable recruitment rate of 6 enrolled per month prior to being disrupted by COVID 19.

**Conclusions:**

A tiered approach to eligibility screening using a hybrid of web-based portals to self-identify and screen for general eligibility followed by a more detailed phone and medical record review allowed the study to use fewer sites and reduce cost. Despite the difficult and narrow population of patients suffering moderate chronic disability from stroke, this strategy produced a viable recruitment rate for this invasive study of intracranially injected neural stem cells.

**Trial registration:**

ClinicalTrials.gov Identifier: NCT03629275

**Supplementary Information:**

The online version contains supplementary material available at 10.1186/s13063-024-07988-z.

## Background

Recruitment of participants is the greatest risk to completion facing most clinical trials, with 20–40% of trials failing to reach the targeted enrollment [1]. This is particularly true of trials of central nervous system (CNS) therapies where protracted trial periods contribute to the 38% longer delays in approval of CNS drugs compared to non-CNS drugs [2]. Stroke trials are particularly susceptible to slow enrollment, both in acute intervention trials where the window for recruitment is very limited [3–5] as well as in chronic disability trials where it may be difficult to reach potential participants since they are not as frequently engaged with the primary stroke center or rehabilitation services [6, 7]. Furthermore, even if there is ongoing contact with rehabilitation centers, these facilities are rarely equipped to conduct interventional research, particularly if this is complex and early phase. Traditional approaches to the problem of recruitment have been to include more sites in the study and extend the recruitment period. This strategy adds substantial cost and time to the trial and produces geographical limits on recruitment leading to disparities in access to trials. Other strategies focusing on participants (i.e., mailings, flyers and phone calls) or comparing types of recruiters (i.e., nurses, coordinators, physicians) have not demonstrated significantly improved success, leaving the question of how best to optimize recruitment largely unanswered [8, 9].

These recruitment challenges have led to the exploration and greater use of Internet-based approaches to trial recruitment [10–12]. Behavioral intervention and survey-based studies were early adopters, leveraging web-based recruitment with social media advertising, and were simple and efficient [13–15]. Other approaches used websites for specific communities to produce registries and provide a portal for self-reported outcomes and data aggregation [16]. An online survey promoted through social media was successful in a trial of Parkinson’s disease with broad inclusion criteria that did not require extensive screening [17]. With these early successes, Internet trials were developed in which the entire study was conducted remotely resulting in semi-automated randomized controlled trials (RCTs) such as the Adaptable aspirin trial [18]. These studies have demonstrated the feasibility and value of using Internet-based recruitment for some clinical trials, including randomized trials, and provided the basis for considering this approach for a surgical intervention trial for chronic stroke disability (PISCES III).

The PISCES III trial was an invasive trial of stereotactically guided intracerebral injection of stem cells for chronic disability due to ischemic stroke [19]. Potential participants were required to have moderate disability as defined by modified Rankin score of 3 or 4 following a qualifying ischemic stroke event at least 6 months but not more than 18 months prior to the investigational treatment. Participants also required sufficient brain tissue to allow injection on the side of the stroke which would require review of prior imaging as part of prescreening when available. Effective prescreening might therefore limit the number of time-consuming imaging and chart reviews required and greatly increase the yield from those reviews. Here, we report on the successful experience using a novel hybrid recruitment approach of a patient-facing portal to self-identify and perform an initial screen for general trial eligibility (tier 1), followed by phone screening and medical records review (tier 2) before a final in-person visit to confirm eligibility and obtain consent.

## Methods

### Overall recruitment strategy

The study design and related procedures have been published elsewhere [19, 20] and are provided as a trial summary in Additional file 1. Briefly, the study was designed to recruit 110 participants randomized to receive stereotactic neurosurgical implantation of stem cells into the striatum ipsilateral to the stroke or sham surgery. To enhance safety and simplify the logistics of shipping and delivering CTX0E03, an effort was made to reduce the number of surgical sites to a small number (6) of high-volume, highly experienced stereotactic neurosurgical centers and ensure a standardized approach for delivery of the investigational product [20]. Participants needed to travel to the surgical sites, but only once during the trial, and efforts were made to have a larger number of assessment sites to allow for shorter and more convenient travel for the baseline and post procedural follow-up visits. The assessment sites (20) were largely selected for their proximity to densely populated areas with relatively high stroke patient volumes and prior stroke research experience. The planned use of alternative strategies such as web-based approaches for identifying, contacting, and screening potential participants allowed for lower overall numbers of sites, but the lower site number limits the effectiveness of traditional site-based recruitment approaches. As a result, a two-pronged approach to study recruitment was formulated and is described here.

### Traditional regional and site-based recruitment

Two strategies were planned for site-based recruitment. Traditional paper flyers and advertisements were prepared for display in clinics, and a study website was created to allow centers to refer their patients to the website for additional information. The link to a screening survey was also provided to the sites so they could assist potential participants with the screening survey. The second strategy involved the use of local registries of stroke patients which are maintained by most comprehensive stroke centers. The registries allowed for identification of patients that had larger strokes and thus likely to have residual disability. The ability to use the registries as a source for recruitment was based on the approval of the local institutional review boards as not all sites were allowed to use this approach to trial recruitment.

### Radio, TV, site-based, flyers and physician referrals

Traditional advertisement material was created by a third-party vendor, Clinical Trial Media (CTM). The advertisements were run at various times early in the recruitment period to increase the visibility of the trial within communities. Flyers were provided to the sites and were sent to stroke clinics associated with the study sites and to sites upon request. All advertising material provided the phone number and information for accessing the patient-facing portal to complete initial screening. Callers were either directed to the portal or administered the portal screen via phone when Internet access or inability to complete the portal survey online was an issue. Participant family members were also completing the surveys and the sites were encouraged to assist any study referrals to facilitate screening and medical records release. Ultimately, all potential participants were funneled through the patient-facing portal for screening.

### Facebook, Google, and YouTube

Internet and social media advertising was run nearly continuously once the content was created. The YouTube video provided an overview of the trial, who qualified for the study, and the overall procedure process. All Internet material directed viewers to the patient-facing portal for screening to establish if inclusion criteria were met.

### Design of screening approach

**Fig. 1:**
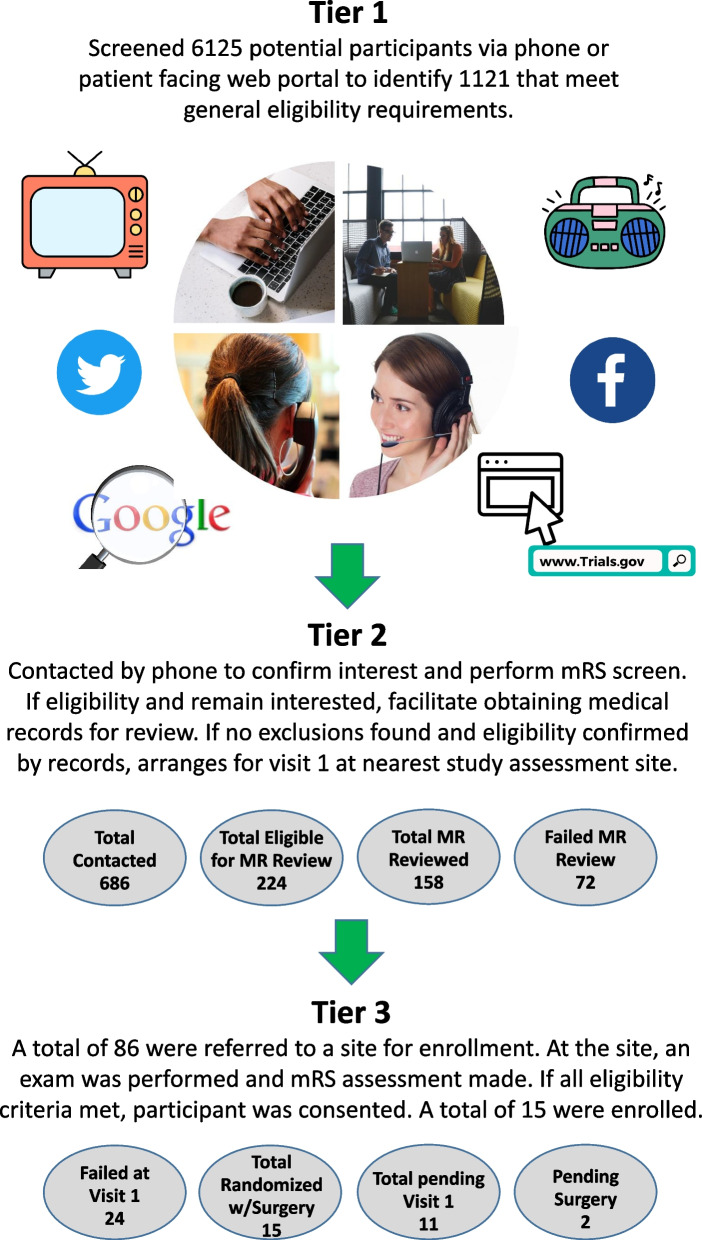
Overall recruitment flow diagram. Multimedia advertising directed potential participants to tier 1 screening via patient facing web portal eligibility survey. Those meeting primary eligibility criteria were referred to tier 2 screening. Standardized phone assessment of modified Rankin score and level of disability were used to determine appropriateness of obtaining medical records to further screen for eligibility. Those appearing to meet the study inclusion and exclusion criteria were then referred to the nearest study assessment site for formal evaluation and consent at visit 1

Figure 1 summarizes the overall flow of the screening approach for the study. All contact and screening processes were approved by a central IRB reviewed protocol and frequently approved again by each participating site’s IRB. The study had relatively complex eligibility requirements which are fully detailed in the parent trial [19]. Briefly, participants needed to meet the inclusion and exclusion criteria provided in Additional file 1. Given the extent of the criteria, we anticipated there would be a high screen failure rate and that many more potential participants would need to be screened to find the few that met trial criteria. This would potentially carry a very high cost to both the sponsor and the assessment sites if all screening was performed in person.

In order to screen large numbers of potential participants for general eligibility, a brief web-based survey was developed that could screen for appropriate time post stroke event, stroke type, and relative level of disability (Additional file 2). The final tier 1 call center phone script and patient-facing portal survey is also provided in Additional file 2. The functional movement questions were based on the need to have some degree of movement in the stroke-affected limbs since this was required in order to be assigned a level of rehab within the Graded Repetitive Arm Supplementary Program (GRASP) therapy program [21]. The survey was built in a commercial platform and hosted by CTM. The data used for this project was derived from CTM reporting of activity at each level of the screening process. Through CTM, the survey could be completed via the website or via phone. Qualifying participants were then referred to the primary central screening center (PCC) at the Duke Clinical Research Institute (DCRI).

The PCC tier 2 screening and chart review process at the DCRI consisted of a phone call to confirm participant interest and willingness to answer some additional screening questions and to confirm eligibility. The screening survey was then repeated by a clinical nurse coordinator along with the phone-based focused Rankin assessment scale that was previously described and validated [22–24]. The development and final version of this screening tool following alignment with the sites is provided in Additional file 2. If the participant screened as eligible, contact information was collected and forms for medical records release were completed and submitted to the health care facility in which they received care for their qualifying stroke. Medical records received were sorted into a review packet consisting of ED notes, history and physical from the admitting service, neurology consult, if available, discharge summary, and all imaging reports and then reviewed by the medical monitor. Any additional documents were scanned and retained digitally in the event study eligibility could not be verified by the medical monitor using the review packet. The medical record review was used to ensure the stroke was of the ischemic type, sufficient brain tissue was left to allow delivery of investigational product, and neurologic deficits and residual disability from the index stroke event were consistent with trial requirements and current patient-reported deficits, date of stroke, and medical fitness for the surgery and study protocol. In the event that all of these elements could not be confirmed by chart review, an additional screening CT was recommended to the assessment site as part of the screening visit. If the chart review confirmed eligibility, then participants were contacted and travel arrangements were made to visit the nearest assessment site for formal screening and consent.

### Tracking and analyses of enrollment

The analyses presented here were conducted on three separate sources of information that were used to track and report on recruitment of participants through the described screening processes. The first source was the CTM tracking metrics for the volume of calls and website traffic that was created as a daily log of screening activity and consisted of the date and time each potential participant was screened and their answers to the screening survey. The second was the PCC screening log. This was used to track contact and phone screening of each potential participant that was screened eligible by CTM and referred on to PCC for further consideration. The final source was the advertising dates, times, and media types running during the recruitment period. The volumes from these sources were aggregated by month and then plotted together to provide a relative yield for each step of the screening process over time. The relative changes in screening volume were aligned by the sponsor with advertising expenditures to determine which forms of media were most effective. We looked at the relative proportions of participants that were screened by phone compared to website and how many completed the screening themselves compared to someone else calling on their behalf. To assess the success of the screening, we looked at the ability of participants to provide information about their level of disability, stroke type, and cause(s) of their stroke via survey and by phone screen compared to the medical records review and first visit screen fail rate. It was the expectation that the extensive prescreening would greatly reduce the site-based screening failures to less than 20%. We also tabulated general self-reported observations about the screened cohort to help better understand the disabled stroke population. Given the very low enrollment in the study prior to closing, formal yield calculations and statistical comparisons were not possible.

## Results

### Overall screening effectiveness

**Fig. 2:**
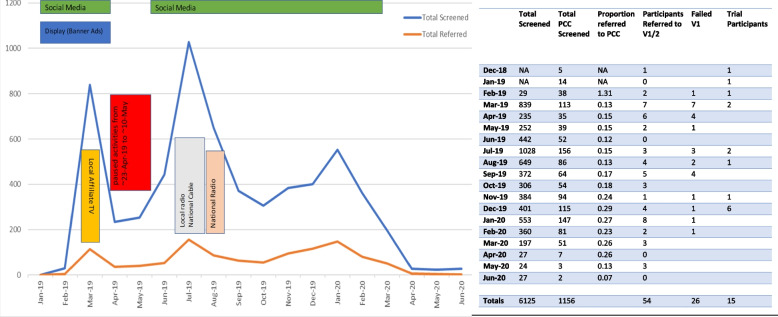
Recruitment activity over the course of the recruitment period of the trial. In the graph, traditional media campaigns produced large spikes in screening activity within the patient-facing portal and call center (tier 1). This also produced slight increases in the number of potential participants referred to tier 2 for screening. Online searching and social media ads were ongoing throughout the recruitment period, nearly continuously, with the exception of a brief period of suspended enrollment during a protocol amendment (red rectangle). The table provides the actual raw numbers of potential participants screened at each level and referred to a site for formal assessment and consent. Abbreviations: *Total Screened* is the total number that completed tier 1 screening, *Total PCC screened* is the total number of potential participants referred to the primary central screening center (PCC) at the Duke Clinical Research Institute, *Proportion Referred to PCC* is the ratio of total referred to PCC to the total screened in that month, *Participants Referred to V1/V2* is the number of participants referred to visit 1 and visit 2 at a study assessment site, *Failed V1* are the number of participants that did not meet inclusion criteria when seen and evaluated at visit 1, *Trial participants* is the number consented and enrolled in the study that month

Figure 2 summarizes the overall screening activity at monthly intervals over the recruitment period of the trial from December 2018 to June of 2020. The data used for this analysis are based on the 16 months of screening performed by CTM. A total of 6125 pre-screens were conducted over the CTM platform. Phone call pre-screens were conducted for 2070 (34%) with the remaining 4055 (66%) being website-based and 18 of those were on the Spanish language website. A total of 5446 (89%) completed the pre-screener survey process, and only 679 (11%) did not. Of those completing the screening, 4290 (79%) were disqualified, and 1156 (21%) resulted in referrals to the central screening center for further consideration.

Following the first level of web survey screening, 1121 potential participants were referred to the PCC with 35 still pending referral to the PCC at the time of study termination. Multiple attempts were then made to contact the referred participants to provide additional details about the study, confirm answers to the web survey to confirm disability, perform phone-based modified Rankin score (mRS) assessment, and then, if still eligible, assist in obtaining medical records for review. The PCC screening team was not able to contact 24% of the referrals (268). Another 9% (101) declined to participate once they understood the trial in more detail. There were 64 potential participants pending contact at the time of trial termination. The screeners conducted a validated phone-based focused mRS assessment with the remaining 686 potential participants, and the results are summarized in Table 1. The phone screen failed 41% of the referrals due to low mRS (mRS < 3). The largest mRS category was 2. The targeted mRS scores of 3 and 4 were 33% of the subjects. An estimated 350 hours were required to perform the tier 2 screening described, with additional 50 hours required for medical chart review by the medical monitor.

**Table 1:** Distribution of modified Rankin scores from phone screen by the PCC

mRS	PCC screen
0	13
1	156
2	256
3	171
4	53
5	37
NA	435
Total referred	1121
Total screened	686

*mRS* modified Rankin score, *PCC* primary central screening center (tier 2)

A total of 224 had medical records requested of which 158 records were ultimately received and 72 participants failed at the medical records review stage leaving 86 to move forward to evaluation at a study site nearest to them. One important observation was the number of participants that passed the eligibility checks and PCC phone mRS screen (459, 67%), and then, despite being previously validated for use by phone [22, 24], failed the mRS assignment at the first visit with the study site (24, 28%). The initial site failure of PCC referrals was close to 50%. As a result, a review of the mRS screening tools was performed as detailed in Additional file 3, and conference calls with site teams were held to determine how best to improve screening accuracy and agreement. From these discussions, it was determined that a clear definition of the target participant was needed, and the mRS categorical definitions provided by Shinohara and colleagues [25] were adopted; the screening tools were unified across all screenings to the simplified modified Rankin score questionnaire [26] with modification to the first question to include the temporal context of 1 week (Additional file 3). Site retraining was performed, and site investigator agreement on the new screening process was obtained. These interventions improved the congruency between the central screening and site-based screening and lowered the final site screen fail rate to 28%.

### Media advertising effects on recruitment

As shown in Fig. 2, active online advertising did result in relatively constant website and call screening volumes. Social media campaigns were ongoing throughout the recruitment period, with the exception of a brief 3-week period from 23 April 2019 to 10 May 2019. Additional TV and radio advertising resulted in notable, albeit transient, increases in screening volume.

Sources for referrals are summarized in Table 2. As intended, the vast majority of the referrals came from Internet sources whether through searches or social media. Facebook and other social media resulted in the largest number of referrals to the PCC for consideration with Internet search in a close second. The Internet searches resulted in finding the trial via ClinicalTrials.gov, local hospital or rehab websites, the trial website, or the YouTube ad for the trial. Of note was the significant volume of referrals from “word-of-mouth” from friends, family, and other medical providers. Television holds a substantial edge over radio and print ads but constitutes less than 10% of the total referral volume.

**Table 2:** Sources of potential participant referrals screened at tier 1

Source of referral	DQ1	PCC referral	Source total	Success ratio
A current PISCES III study clinic	10	9	19	0.47
Another doctor or rehabilitation specialist or institution	125	44	171	0.26
Facebook, Instagram, or other social media	1877	541	2397	0.23
Hospital or rehabilitation website	97	29	126	0.23
Radio ad	27	7	34	0.21
Google or other search engine	1425	305	1761	0.17
Newspaper or print flyer	11	2	13	0.15
ClinicalTrials.gov	13	2	15	0.13
TV ad	333	47	380	0.12
Friend or family referral	129	15	144	0.10

*DQ1* disqualified by tier 1 survey, *PCC* primary central screening center (tier 2). *Source total* is the total number of referrals attributed to the source; *Success ratio* is the number of enrolled participants attributed to that source divided by the Source total

To better compare the effectiveness of the various referral sources, we calculated the referral success rate as the ratio of PCC referrals to the total number contacted by the given source (Table 2 success ratio column). Internet searching produced a combined success of 0.53. PISCES III clinics and another doctor or rehabilitation specialist had the next highest success rates of 0.47 and 0.26 respectively. Facebook and other social media came next with success ratio of 0.23. While radio advertisements did not produce a large number for referrals overall (*n* = 34), the referral success rate was 0.21 which was much higher than any of the other remaining referral sources.

### Characteristics of the respondents

To determine if the screening tool was effective at identifying the target stroke cohort and not inappropriately excluding too many potential participants, we examined the self-reported stroke type, stroke causes, and level of disability in those screened. Of those undergoing the initial level of screening, 12% self-reported as having hemorrhagic stroke, 70% as ischemic, and 18% were unsure of their stroke type (Table 3). Most were not sure of the primary stroke risk factor, although hypertension was the most frequently cited cause in all three groups. Ischemic stroke patients also frequently reported blockage of blood flow in the neck as well as atrial fibrillation and blood clots from the heart or legs as the cause for their stroke.

**Table 3:** Self-reported stroke type and cause for stroke

Reported cause	Hemorrhagic stroke	Ischemic stroke	Unknown type
Atrial fibrillation	23	271	33
Blood clot from heart	7	242	8
Diabetes	30	274	115
Hypertension	270	821	251
Blockage of blood flow in neck	30	744	60
Related to heart attack	9	67	19
Blood clot from leg or other body region	24	324	29
Related to procedure	31	153	42
Not sure	330	1449	595
Total	754	4345	1152
Proportion of total	0.121	0.695	0.184
Proportion not sure of cause	0.438	0.333	0.516
Less than 3 months ago	43	310	91
Between 3 and 6 months ago	41	384	94
Between 6 and 12 months ago	57	448	106
Between 12 and 23 months ago	63	561	160
More than 23 months ago	478	2053	557
Totals	682	3756	1008

A total of 5445 potential participants self-reported on their ability to move the stroke affected upper limb at the shoulder, wrist, and finger (Table 4). More respondents reported being able to shrug their shoulder (4181) than move their wrist (3488) or fingers (2702). Of those that could shrug their shoulder, most (3079, 74%) could also move their wrist and many could move their finger (2486, 59%) with just over half reporting they move all 3 (2260, 54%). Of those not able to shrug their shoulder (1264), it was uncommon to still be able to move at the wrist (395, 31%) or the finger (203, 16%). Of the 2260 respondents that were able to move all three joints, over half (1271, 56%) reported feeling disabled, and overall 3918 (72%) of the 6125 respondents reported feeling moderately or severely disabled. Only 28% (1520) reported mild or no deficits and were independent (Table 4).

**Table 4:** Self-reported upper extremity function and perceived disability

(*n* = 5445)		Yes	No
Able to move	Shoulder (S)	4181	1264
	Wrist (W)	3488	1956
	Fingers (F)	2702	2743
	S + W	3079	2366
	S + F	2486	2959
	S + W + F	2260	3185
	Feel disabled	3918	1527
	No/mild deficits	1520	3925
	S + W + F feel disabled	1271	4174

### Summary of yields

Yield was defined as the proportion of participants that were referred to the PCC by the web-based screening survey (tier 1 yield) and the proportion of patients screened by the PCC that were referred to a site for review and consent (tier 2 yield). We also calculated the overall yield of randomized patients from the total screened by CTM. The tier 1 yield was 1121 referrals from 6125 CTM contacts or 18.3%. The tier 2 yield was 86 of 1121 or 7.6% of the PCC referrals. The overall yield for the recruitment approach was 17 confirmed participants and another 11 pending visit 1 and consent for the study for an estimated 23–25 total study participants, assuming 6–8 of the 11 qualify and agree to participate. This represents 0.4% of the screened subjects and is a yield of 1 participant for every 245 screened.

## Discussion

Recruitment of participants remains a major challenge facing most clinical trials, with 20–40% of trials failing to reach the targeted enrollment, compromising study feasibility [1]. We report our experience using a patient-facing web-based portal approach to recruitment and screening for an invasive neurosurgical trial to treat moderate chronic stroke disability. The program successfully connected with the targeted population, and initial digital screening effectively excluded most ineligible participants. The advertising was effective at driving potential participants to the web-based patient-facing portal for screening, with social media advertising and Internet searching advertisements being the most effective at generating referrals to the portal.

When this recruitment strategy was initially developed in 2014, it had never been used for an intervention other than behavioral or survey-based studies [8, 10–13, 27]. Since the termination of the trial and preparation of this manuscript, another medical treatment trial has used a similar web-based screening and enrollment approach [27], although PISCES III remains the first invasive surgical trial to use this approach. It was specifically chosen to allow a reduced number of surgical sites for the trial to reduce costs [11], to facilitate the logistics of shipping and management of CTX0E03, and to leverage web recruitment to reach more potential participants since most would not necessarily be closely affiliated with an academic or rehabilitation center which impairs effective traditional site-based recruitment. However, one of the main concerns was that older stroke patients would not be engaged with the Internet or social media and the approach would not successfully connect to the targeted population. However, this was clearly not the case. Not only are older people increasing their use of the Internet and social media sites like Facebook [28], we found that their children, caregivers, and younger family members were actively searching and seeking potential trials and therapies for them. Our data that digital searches and advertising were the most common sources of referrals and more traditional advertising led to significant increases in traffic to the portal screening site. This approach also demonstrated the importance of, and real-world use of, trial registries like clinical trials.gov informing the public on available trials.

Our approach allowed the screening of 6125 potential participants in 16 months and led to the ultimate enrollment of 15 participants from 1121 referred for tier 2 screening. The trial had features that have been found to be high risk for failure due to enrollment including high number of eligibility criteria, an early phase trial, and small number of trial sites [1]. However, in the final month of the trial, there were 6 participants pending randomization and surgery with an additional 11 pending tier 2 screening. If the 6/month enrollment rate, augmented by site-based recruitment that was just being initiated, had continued, we would anticipate successful enrollment for the study within 16 months. Unfortunately, the onset of the COVID-19 pandemic led to premature termination of the study.

Our tier 1 screening portal identified 1156 (21%) for subsequent, more detailed tier 2 screening and review. It is important to note that about 1/3 of tier 1 screens were completed by phone, demonstrating a need to continue to support this option for potential participants. We found that 89% of the screening surveys were completed, which is higher than other reported online survey screens used for trials [10–13, 27]. This may be indicative of a highly motivated population that is seeking intervention for their chronic disability. However, this result is in contrast to our finding that 24% of these selected referrals to tier 2 screening could not be contacted, and the reasons for this remain unclear since they were successfully screened by completing the survey at tier 1. Further studies are needed to determine the reasons for subsequent contact failure rate of 24%, which may include multiple survey attempts, family members exploring the study on someone's behalf with limited information, or post-screen health issues or death.

Despite using a phone validated mRS assessment tool [22] we initially saw a significant discrepancy (50% screen fail rate), between the phone-based mRS and the in-person site mRS. This was generally not the result of participants intentionally misrepresenting their level of disability, although this did occur (2 participants). It was largely reflective of differences in the participants’ perception of their level of disability and objective study personnel that assigned scores. It also reflects the poor sensitivity of the mRS to upper extremity dysfunction. We addressed this through site training, establishing a common interpretation for some of the screening questions and providing a common scenario for reference. These changes were reflected in the modified scripted language used in the phone screening tool (Additional file 3) and resulted in reduction of site screen failures (28%). Our results indicate the need for skilled site-base coordinators to perform the screening to ensure required inclusion/exclusion criteria are met. The most common mRS in our tier 2 cohort was 2 suggesting the need for future patient preference studies to better characterize the trade-offs patients are willing to make in terms of risks of medical and surgical interventions to improve their function since these potential participants were apparently willing to undergo invasive brain surgery to treat their perceived level of disability which was mild and would classify them as “Independent.”

Our analysis of the recruitment approach has some limitations. While site-based recruitment was planned and in the process of being operationalized, it was never initiated so it is unknown how it may have contributed to overall monthly recruitment rates or how it compared to the web-based approach. We were also not able to perform any financial analysis due to contractual limitations. Finally, the study was terminated by the COVID-19 pandemic, and so the full functional rate of recruitment may not have been attained and could impact the relative importance or contribution of the different advertising pathways contributing to recruitment.

## Conclusions

A patient-facing web-based portal screening approach was effective and identified the target population of chronically disabled stroke survivors for this invasive surgical trial. We were not able to assess costs or the impact of additional site-based recruiting, but at the time of trial termination, we had achieved a viable enrollment rate for the trial. Motivation was high with most surveys completed once started; however, differences in perceived disability and the objective scoring using mRS resulted in significant in-person screen failures even after two-tiered screening and medical record review. The in-person screen failures can be mitigated by aligning clinical context among all the sites.

### Supplementary Information

**Additional file 1.** Full Inclusion/Exclusion criteria for PISCES III

**Additional file 2.** Screening Website For Web-Based Pre-Screening

**Additional file 3.** Summary of Analysis of mRS Approaches and Agreement

## Data Availability

Data reported in this manuscript is available upon written request.

## Abbreviations

CNS: Central nervous system
CTM: Clinical Trial Media
GRASP: Graded Repetitive Arm Supplementary Program
mRS: Modified Rankin score
PCC: Primary central screening center
RCT: Randomized controlled trial
