# Supplementary material for: Experience with a hybrid recruitment approach of patient-facing web portal screening and subsequent phone and medical record review for a neurosurgical intervention trial for chronic ischemic stroke disability (PISCES III)
Source: Trials. 2024 Feb 28;25:150. doi: 10.1186/s13063-024-07988-z (PMC10900735; doi:10.1186/s13063-024-07988-z)
Supplement: Supplementary file 1 — Additional file 1. Full Inclusion/Exclusion criteria for PISCES III [file 13063_2024_7988_MOESM1_ESM.pdf]

Appendix 1: Full Inclusion/Exclusion criteria for PISCES III

Inclusion	Exclusion
1. Written informed consent or witnessed informed consent in the event that the subject is unable to sign informed consent.<br>2. Ischemic stroke that includes the supratentorial region occurring within 6 to 12 months of the time that surgical intervention will be performed (Qualifying Stroke Event)<br>3. Aged between 35 and 75 (inclusive)<br>4. Qualifying Stroke Event must be confirmed by CT or MRI<br>5. Must have current Moderate or Moderately Severe disability as measured by modified Rankin Score = 3 or 4 due to the Qualifying Stroke Event<br>6. Must have some residual upper limb movement as defined by the GRASP manual e.g. ability to actively shoulder shrug against gravity and wrist extension (palpable by the investigator; or visible lift of the fingers with hand on table)<br>7. Must have sufficient cognitive and language abilities to comprehend verbal commands for study assessments<br>8. No medical conditions that would preclude neurosurgery with appropriate preparation and management.<br>9. Sufficient putamen, globus pallidus or caudate nucleus volume to enable delivery of the CTX0E03 DP<br>10. Able to attend study related Visits and complete any diary, telephone or questionnaire assessments<br>11. Females of childbearing potential (FOCBP), (or within 2 years of last menstrual cycle) must have a confirmed negative pregnancy test at time of treatment and agree to use two r reliable methods of contraception (e.g. oral contraceptive and condom, intra-uterine device and condom, diaphragm with spermicide and condom) for six months following surgery<br>12. Sexually active males with partners who are FOCBP must be willing to use a reliable method of contraception (e.g. barrier and spermicide or as described above) for six months following surgery.	1. Permanent disability corresponding to a Modified Rankin Score of >1 prior to the Qualifying Stroke Event.<br>2.Stroke due to hemorrhage or related-to connective tissue disorder, congenital disorder of the cerebral vessels or a disorder of thrombosis Subjects with atrial fibrillation as a suspected cause of stroke are NOT excluded.<br>3. Neurosurgical pathway obstructed by vascular malformation or cavity<br>4. History of neurological or other disease resulting in significant functional impairment<br>5. Any contraindications to either CT scan or MRI<br>6. Inability to stop or transition off valproic acid or other demethylating agents or HDAC inhibitors for 1 week before and 4 weeks after treatment with CTX0E03 DP<br>7. Use of selective serotonin reuptake inhibitors (SSRI), unless the subject is on a stable dose that has been started at least 2 months before screening<br>8. Use of antispasticity medications (excluding oral antispasticity medications if they have been taken regularly for at least four months prior to treatment with CTX0E03)<br>9. Inability to discontinue anticoagulation therapy<br>10. Severe comorbid disorder that has reasonable likelihood of limiting survival to less than 24 months.<br>11. History of malignant disease within the last 5 years, (excluding benign tumors such as non-melanoma skin cancer, cervical carcinoma in situ, superficial bladder cancer)<br>12. Any history of primary or secondary brain malignant disease.<br>13. Previous participation in a cell-based therapy study at any time or in any other study involving an investigational product or rehabilitation study within the last 30 days<br>14. Clinically significant laboratory values, including positive Class I HLA antibodies specific for CTX0E03, during screening<br>15. Inability to adhere to the study post-surgery upper limb standard PT regimen e.g. excessive spasticity or pain<br>16. Planned initiation of any other new physical therapy regimen within 6-months post-treatment<br>17. Any other conditions that, in the opinion of the investigators, would preclude safe or effective participation

CT (computed tomography); CTX0E03 (investigational product); FOCBP (female of child bearing potential); GRASP (graded repetitive arm supplementary program); HDAC (histone deacetylase inhibitor); HLA (human leukocyte antigen); MRI (magnetic resonance imaging); PT (Physical Therapy)
