# Supplementary material for: Experience with a hybrid recruitment approach of patient-facing web portal screening and subsequent phone and medical record review for a neurosurgical intervention trial for chronic ischemic stroke disability (PISCES III)
Source: Trials. 2024 Feb 28;25:150. doi: 10.1186/s13063-024-07988-z (PMC10900735; doi:10.1186/s13063-024-07988-z)
Supplement: Supplementary file 2 — Additional file 2. Screening Website For Web-Based Pre-Screening [file 13063_2024_7988_MOESM2_ESM.pdf]

Appendix 2

Screening Website
For Web-Based Pre-Screening

Sponsor:	ReNeuron	Document:	ReNeuron_PiscesIII_Stroke_Webscreener Script_V 2.3_9.26.19_US English_CLEAN.docx
Study:	PISCES III Stroke	Version:	V2.3
		Status:	Protocol Amendment
Design Format:	.2	URL:	www.PISCES3.org

Element Name<br>*[no IRB review]*	Content for IRB Review<br>*[Text in blue and graphics are visible to callers]*	Programming<br>*[no IRB review]*
	**Cookies Statements**	
Cookies Statement	**Your choice regarding cookies on this site**<br>We use cookies to optimize site functionality and give you the best experience. Necessary cookies enable core functionality. The website cannot function properly without these cookies, and can only be disabled by changing your browser preferences.<br><br>For more detailed information on the cookies we use, please check our Privacy Policy.<br><br>By continuing to access this website you are giving us consent to collect cookies.<br><br>[Accept]	**Privacy Policy** links to Screen 11
	**General Design Elements & Graphics** (All Screens) (Original text/format from PISCES3.ORG)	
Banner (Original text/format from PISCES3.ORG)	A CLINICAL RESEARCH STUDY of stem cells for patients with ONGOING DISABILITY following STROKE	

Footer<br>(Original text/format from PISCES3.ORG)	PISCES III<br>PHASE IIb INVESTIGATION OF STEM CELLS IN STROKE<br>ABOUT<br>PARTICIPATE<br>FAQ<br><br>ReNeuron<br>Privacy Statement Legal Notice<br>Duke Clinical Research Institute | Durham, North Carolina, USA	**About** links to Screen A<br>**Participate** links to Screen B<br>**FAQ** links to Screen C<br><br>**Privacy Statement** links to Screen 11<br>**Legal Notice** links to Screen 12

**Screen #1: Home** (default) (Original text/format from PISCES3.ORG)	
Call 1-844-707-8336 to see if you pre-qualify<br><br>ABOUT PARTICIPATE FAQ<br><br>PISCES III is a clinical research study to determine whether stem cells injected into a damaged area of the brain can improve function in people with ongoing disability following an ischemic stroke (a stroke that occurs when blood flow to the brain is blocked, such as by a clot). This study is open to U.S. and U.S. territory participants only.<br><br>[Take The Eligibility Survey]<br><br>Life changed that day...<br>Some patients who have had a stroke make a complete or nearly complete recovery. Other patients do less well and are left with ongoing disability. This study is for patients between 35 and 75 years of age, who have limited movement of their arms and/or legs 6–24 months after a stroke.<br><br>Study Overview	**About** links to Screen A<br>**Participate** links to Screen B<br>**FAQ** links to Screen C<br><br>**Take the Eligibility Survey** links to Screen 1a

The purpose of the PISCES III study is to find out if a study drug, which is made from stem cells, will provide any improvement in the arm and/or leg function affected by an ischemic stroke. The name of the study drug is CTX0E03 DP, and it is injected into the brain during surgery. The study will assess the effectiveness of CTX0E03 DP to change the degree of dependency and disability of study participants from baseline, as well as measure a number of safety parameters.<br><br>[Learn More]	**Learn More** links to Screen A
**Screen #A: About** (Original text/format from PISCES3.ORG)	
A STUDY OF STEM CELLS IN STROKE ABOUT<br><br>PISCES III PHASE IIb INVESTIGATION OF STEM CELLS IN STROKE<br>ABOUT PARTICIPATE FAQ<br><br>LEARN MORE OR JOIN THE STUDY<br><br>About the Study<br>PISCES III is a clinical research study to determine if a study drug, which is made from stem cells, will help improve function in people between 35 and 75 years of age, who have limited movement in their arms and/or legs 6–24 months after having an ischemic stroke. Ischemic stroke is the most common type of stroke, and occurs when blood flow to the brain is blocked, such as by a clot. This study is open to U.S. and U.S. territory participants only.<br><br>The drug being studied is called CTX0E03 DP. The study drug is made of stem cells and is injected directly into the brain during surgery. The study will assess the effectiveness of CTX0E03 DP to change the degree of dependency and disability of study participants from baseline, as well as measure a number of safety parameters. CTX0E03 DP has been studied previously in 34 people who have had an ischemic stroke.<br><br>Patients who meet all of the eligibility criteria and enroll will be placed randomly in one of two groups: One will receive the stem-cell injection into the brain during a surgical procedure; the other group will not receive stem cells during a surgical procedure. You will learn which group you are assigned to once the study has been completed.	**About** links to Screen A<br>**Participate** links to Screen B<br>**FAQ** links to Screen C<br><br>**Learn More** links to Screen A<br>**Join the Study** links to Screen B

<table>
<tr><td>All participants will be provided and expected to complete a 12-week physical therapy program and monitored for 12 months. The sponsor will also be conducting a second study which you will be asked to participate in to follow your long-term progress.

If you would like to speak to a study nurse to see if you pre-qualify, please call 1-844-707-8336.

About ReNeuron
This study is sponsored by ReNeuron, a leading, clinical-stage cell therapy development company. Their primary objective is the development of novel stem cell therapies targeting areas of significant unmet or poorly met medical need.

ReNeuron has used its unique stem cell technologies to develop cell-based therapies for significant disease conditions, where the cells can be readily administered “off-the-shelf” to any eligible patient without the need for additional immunosuppressive drug treatments.

About Duke Clinical Research Institute
Duke Clinical Research Institute (DCRI) is the center coordinating the study on behalf of ReNeuron. The mission of DCRI is to develop and share knowledge that improves the care of patients around the world through innovative clinical research.

[about] [participate] [faq]</td><td>About links to Screen A
Participate links to Screen B
FAQ links to Screen C</td></tr>
<tr><td colspan="2">Screen #B: Participate (Original text/format from PISCES3.ORG)</td></tr>
<tr><td>A STUDY OF STEM CELLS IN STROKE PARTICIPATE

PHASE IIb
INVESTIGATION
OF STEM CELLS
IN STROKE
PISCES III

ABOUT PARTICIPATE FAQ

LEARN MORE OR JOIN THE STUDY

How to Participate
1. Complete a brief <u>eligibility questionnaire</u> through our secure patient portal. If you're eligible for the study, the Duke Clinical Research Institute call center will contact you for more information. If you would like to speak to a study nurse instead, please call 1-844-707-8336.
2. Complete additional screenings at an assessment center to confirm eligibility.</td><td>About links to Screen A
Participate links to Screen B
FAQ links to Screen C

Learn More links to Screen C
Join the Study links to Screen 1a</td></tr>
</table>

3. Complete pre-surgical assessments and undergo a surgical procedure at a surgical center.<br>4. Perform physical therapy exercises daily at home for 12 weeks, and attend follow-up visits at an assessment center every couple of months for one year.<br><br>Study Sites<br>This study is open to U.S. and U.S. territory participants only. The map below shows the locations where study sites may open. If there is not currently a site planned for your area, you still have the option to voluntarily provide your information.<br><br>If you need to travel long distances for your visits, the related costs will be reimbursed for you and your caregiver. This may include mileage, airline fees, hotel stays, and meals. Assistance with travel planning can be provided by the surgical site staff.<br><br>[GOOGLE MAP]<br><br>[about] [participate] [faq]	**Eligibility questionnaire** links to Screen 2<br><br>**Provide your information** links to Screen 2<br><br>**About** links to Screen A<br>**Participate** links to Screen B<br>**FAQ** links to Screen C<br><br>Include Key with Google Map:<br>Key:<br>Assessment Site: Red Pin<br>Surgical Site: Blue Pin<br>Assessment and Surgical Site: Purple Pin<br><br>Site information to show:<br>full institution name, address, city, zip
**Screen #C: FAQ** (Original text/format from PISCES3.ORG)	
A STUDY OF STEM CELLS IN STROKE FAQ<br><br>PISCES III<br>PHASE IIb INVESTIGATION OF STEM CELLS IN STROKE<br><br>ABOUT PARTICIPATE FAQ	**About** links to Screen A<br>**Participate** links to Screen B<br>**FAQ** links to Screen C

LEARN MORE OR JOIN THE STUDY<br><br>Who can participate in this research study?<br>This study is recruiting approximately 130 adults 35 to 75 years old with ongoing disability stroke. There are additional criteria that are included in the questionnaire on this website (www.PISCES3.org/questions.aspx) and some that are reviewed by the study team at the study center at the first visit. You must meet all criteria to be able to participate.<br><br>What is being tested in this study?<br>The study drug, CTX0E03 DP, is made from stem cells and is injected in the brain during surgery. The study drug and the equipment being used to inject the stem cells are experimental, meaning they have not been approved for use by the U.S. Food and Drug Administration (FDA). They are being studied in PISCES III as a possible treatment to improve movement in arms and legs after a stroke. The study drug has been studied previously in 34 people who have had an ischemic stroke.<br>Is there a placebo?<br>**Current Protocol Re-Phrasing:**<br>If you qualify for the study, you will be randomly assigned to either the study medication or a placebo group. If you are assigned the study medication, you will receive the stem-cell injection into the brain during a surgical procedure; if you are assigned to the placebo group, you will only receive a surgical procedure with no injection. Neither you or the study staff will know your assignment.<br>**Future Protocol Re-Phrasing:**<br>If you qualify for the study, you will be randomly assigned to either the study medication or a placebo group. If you are assigned the study medication, you will receive the stem-cell injection into the brain during a surgical procedure; if you are assigned to the placebo group, you will only receive a surgical procedure with no injection. Neither you or the study staff will know your assignment. There is a 66% chance of receiving the investigational procedure, and a 33% chance of receiving a surgical procedure with no injection.<br><br>What are stem cells?<br>Stem cells are cells that can develop into specialized cells or can split to produce more stem cells. In many tissues they act as an internal repair system. The study drug stem cells were made from a single sample of fetal brain tissue that was voluntarily donated to research following a legal termination of pregnancy. These fetal stem cells were genetically modified so that large amounts of stem cells could be produced and tested as a potential therapy for stroke.<br><br>How many people will take part?<br>Approximately 130 subjects will be involved in this study in the United States at approximately 30 centers. See where the study sites are located.<br><br>How do I know if I'm eligible?	**Learn More** links to Screen A<br>**Join the Study** links to Screen B<br><br>**Study sites** links to Screen B<br><br>**Online questionnaire** links to Screen 2<br><br>**Eligibility questionnaire** links to Screen 2

This study is open to U.S. and U.S. territory participants only. You will first need to complete an online questionnaire that will ask you details about your stroke, including any remaining disabilities you may have. If you are eligible based on the answers you provide, the DCRI will complete a review of your medical records (you will need to complete a medical records release form authorizing the DCRI to receive a copy).<br><br>If after the review you are still found to be eligible for the study, an on-site visit will be scheduled at one of the assessment centers. You will need to undergo some tests to ensure you are eligible for the study.<br><br>To get started, complete this eligibility questionnaire.<br>If you would like to speak to a study nurse to see if you pre-qualify, please call 1-844-707-8336.<br><br>How long will I be in this study?<br>Your participation in the study will last a little over one year. You can choose to stop taking part in the study at any time without penalty or loss of any benefits to which you are entitled. During the course of the study you will be asked to participate in a second study to follow your long-term progress.<br><br>How many visits are there?<br>Participation includes up to 11 visits to a study center.<br><br>What about my current medications?<br>Do not discontinue any medication unless you are advised to do so by the study center staff or your primary care physician.<br><br>What about compensation?<br>All costs related to your travel, meals, and any overnight hotel stays will be paid for you and, if necessary, your caregiver. Assistance with travel may be arranged if you need to travel long distances to get to the assessment center or the surgical center.<br><br>Do I need health insurance to participate?<br>No, health insurance is not needed in order to participate.<br><br>Where are the study centers located?<br>The study centers are located in multiple states within the United States. If there is not a site near you open, we can arrange travel for you and, if necessary, your caregiver. You can see active study centers by clicking here.<br><br>Why participate in PISCES III?<br>If you decide to participate, you will be an important part of the research team and have a vital role in advancing medical knowledge and understanding of stroke therapy.	**About** links to Screen A<br>**Participate** links to Screen B<br>**FAQ** links to Screen C

Who is funding this study?<br>Funding for PISCES III comes from ReNeuron, a leading stem-cell therapy development company based in the United Kingdom. ReNeuron's primary objective is the development of novel stem cell therapies targeting areas of significant unmet or poorly met medical need.<br><br>Why was I disqualified?<br>Research studies are designed in specific ways to test the study medication for safety and effectiveness. One or more of the answers that you provided were outside of the guidelines for this study. This does not mean you will not qualify for different research studies.<br><br>[about] [participate] [faq]	
**Screen #1a: Confidentiality** (doiqualify.aspx)	
**PISCES III Intake Questionnaire**<br><br>**Confidentiality**<br>Thank you for your interest in the PISCES III study. The purpose of this questionnaire is to help determine if you may be eligible for further pre-screening to participate in the PISCES III study. This is the type of information we will collect and store about you in this questionnaire:<br>Name and contact information<br>Stroke information, including any remaining disabilities, and other simple questions related to your medical history<br><br>The data you provide will be digitally stored on behalf of the research project in the United States in a HIPAA compliant data center and/or on a highly secured server at Duke University Health System in the United States. Data relating to your pre-screening answers will be shared with the sponsor of this study (ReNeuron), who is located in the UK. Your information may be shared with patientprimary, a patient support service that will facilitate (or handle) all planning and payment for your and a caregiver's travel and related expenses to/from site, if you qualify for the study. You may modify or have the data removed at any time by calling us at 1-844-707-8336 and we can assist you. You can also view our privacy policy any time at Privacy Policy.<br>Call the PISCES III team at 1-844-707-8336<br>Email the PISCES III team at PISCES3@duke.edu<br><br>**Consent**<br>By clicking on the "Yes I Agree" button below you consent to participate in the PISCES III Intake Questionnaire as detailed above. If you do not wish to participate please click the "No I Disagree" button. You may stop the registration process at any time. If you choose to stop, we will not use any of the information you have provided.<br>(Registration takes approximately 10 minutes to complete.)	**Privacy Policy** links to Screen 11<br><br>**Yes, I Agree + SUBMIT:** Continue to Screen 2<br>**No, I Disagree + SUBMIT:** Route to Screen #8: DQ/ /No Interest Thank You Text (nointerest.aspx)

○ Yes, I Agree<br>○ No, I Disagree<br><br>[SUBMIT]		
**Screen #2: Pre-Screening Questions** (questions.aspx)		
Pre-Screening Questions<br>Your information will not be sold or shared with third parties for their own separate use.		
Question Label: Source/1	How did you hear about the PISCES III website and study? (Choose one)<br>○ **A current PISCES III study clinic (Please provide the Study Center or Site ID as provided by the study center. It is a 2 digit number)** Text response<br>○ **Another doctor or rehabilitation specialist or institution (Please provide the name of referring doctor or therapist and location (example: Dr. John Smith, Duke Medical Center))** Text response<br>○ **Facebook, Instagram, or other social media**<br>○ **Google or other search engine**<br>○ **Hospital or rehabilitation Website**<br>○ **Other (Please specify)** Text response	Sources subject to change<br><br>Informational only
Question Label/Number: DOB/2	What is your date of birth?<br>Month ▼ Day ▼ Year ▼	**Minimum Age:** 35<br>**Maximum Age:** 75<br>DQ: <35<br>DQ: >75
Question Label/Number: US Resident/3	Do you currently reside in the United States or one of the United States territories?<br>○ **Yes**<br>○ **No**	**Yes**<br>**No:** DQ
Question Label/Number: Most recent stroke (months)/4	How many months ago was your most recent stroke?<br>○ **Less than 3 months ago**<br>○ **Between 3 and 6 months ago**<br>○ **Between 6 and 12 months ago**<br>○ **Between 12 and 23 months ago**<br>○ **More than 23 months ago**	**Less than 3 months ago:** DQ<br>**Between 3 and 6 months ago**<br>**Between 6 and 12 months ago**<br>**Between 12 and 23 months ago**<br>**More than 23 months ago:** DQ

Question Label/Number: Most recent stroke (date)/5	When was your most recent stroke? (if unknown, please provide best estimate)<br><br>**Month ▼** **Day ▼** **Year ▼**	Month (dropdown)<br>Day (dropdown)<br>Year (dropdown, 2018 - 2023)<br>Options include an "Unknown" response
Question Label/Number: # of strokes/6	How many stroke(s) have you had?<br><br>○ **1**<br>○ **2-3**<br>○ **4 or more**	Informational<br><br>**1**<br>**2-3**<br>**4 or more**
Question Label/Number: Type of stroke (recent)/7	What type of stroke was your most recent stroke? (Choose one)<br><br>○ **Ischemic (blockage of blood flow to the brain, such as from a blood clot)**<br>○ **Hemorrhagic (bleeding into the brain)**<br>○ **Not Sure**	**Ischemic (blockage of blood flow to the brain, such as from a blood clot)**<br>**Hemorrhagic (bleeding into the brain):** DQ<br>**Not Sure**
Question Label/Number: Cause of stroke(s)/8	Do you know the cause for your stroke(s)? (Select all that apply)<br>□ **Blockage of blood flow in neck**<br>□ **Related to procedure**<br>□ **Related to heart attack**<br>□ **Atrial fibrillation**<br>□ **Blood clot from leg or other body region**<br>□ **Blood clot from heart**<br>□ **Diabetes**<br>□ **Hypertension**<br>□ **Not sure**	Informational
Question Label/Number: Continue to have physical effects due to stroke/9	Do you continue to have physical effects/deficits due to your stroke? (Pick one that best describes you):<br>○ **I have no deficits.**<br>○ **I have mild deficits, but am able to do all activities and care for myself.**<br>○ **I have modest deficits that prevent me from doing all activities of daily living. For example, I need help with bathing or dressing, but I am able to walk without help; I am able to walk with assistance (i.e. another person and/or an assistive device)**<br>○ **I have significant disability. For example, I cannot walk without help; I am not able to get out of bed.**	**No deficit:** DQ<br>**Mild**<br>**Modest**<br>**Significant**
Question Label/Number: Able to shrug shoulders/10	Sometimes symptoms/deficits due to stroke are on one side of your body more than the other. Thinking about the side of your body with the most symptoms/deficits:<br><br>Please scroll down to view all options and select "Yes" or "No" for each option.	

	Can you shrug your affected shoulder?<br>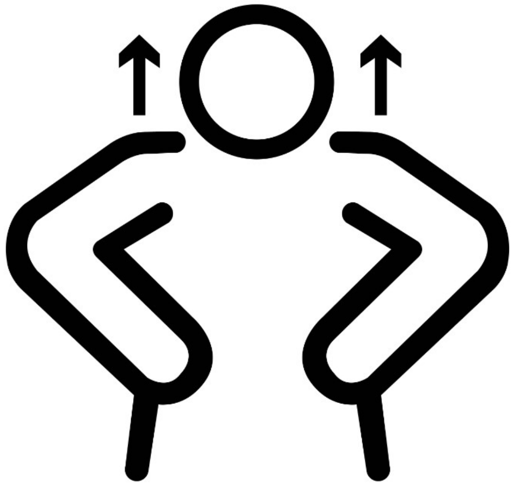<br>○ **Yes**<br>○ **No**	**Yes**<br>**No:** DQ
Question Label/Number: Palm down, able to lift fingers/11	With your affected palm down on a table, can you start to lift your fingers?	

	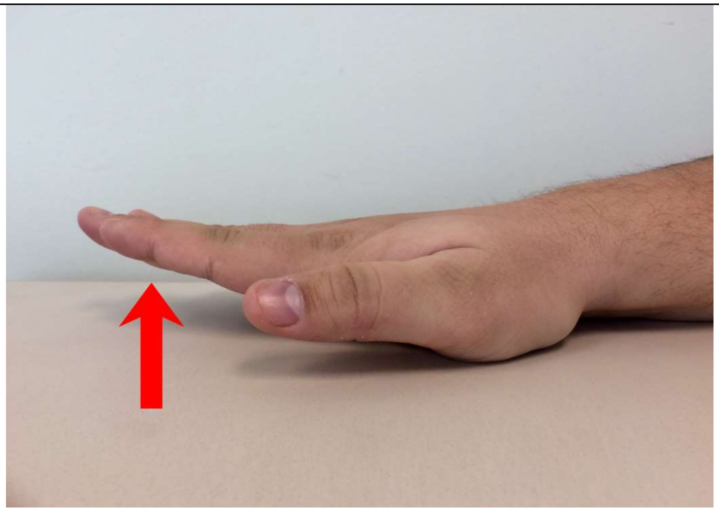<br>○ **Yes**<br>○ **No**	**Yes**<br>**No**
Question Label/Number: Able to make stroke affected wrist move/12	Hold your stroke affected hand with your unaffected hand and try to make your stroke affected wrist move. Can you feel or see it move?<br>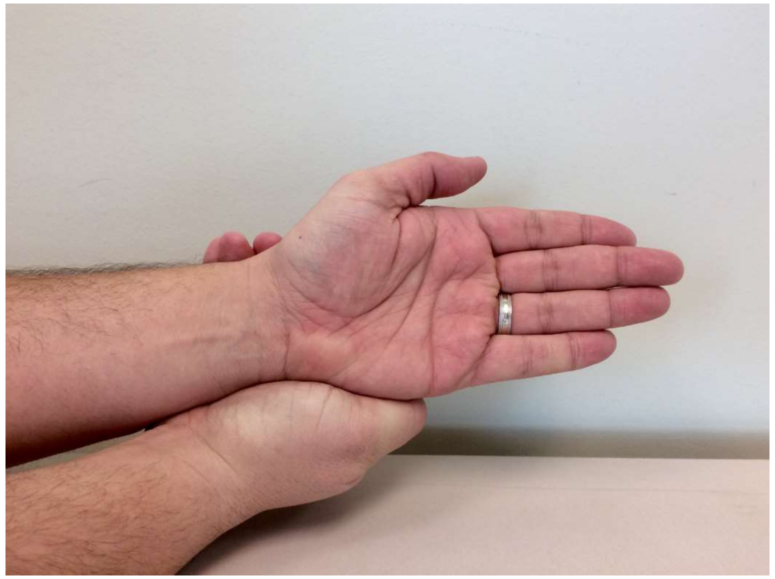<br>○ **Yes**<br>○ **No**	**Yes**<br>**No**

	[Submit]	**Submit:** if any DQ response is selected: Route to Screen 6<br>Custom DQ Programming for combination of Q11 (No) + Q12 (No) = DQ<br><br>**Submit:** All questions answered + No DQ responses: Continue to Screen 4

**Screen #3: Site Locator Text** (sitelocator.aspx) – **NOT APPLICABLE**	
	**Site Locator Screen**<br>Auto select default site for every visitor.

**Screen #4: Pre-Qualified Contact Information Text** (qualifycontact.aspx)			
**Submit Your Contact Information**<br>Based on the answers provided, you qualify for further pre-screening for the PISCES III study.<br>Please note that additional screening is required by the PISCES III team to qualify you for this study. Only the study center staff can determine if you are suitable for this study.<br>Study center locations and travel will be discussed with you by phone once you submit your information<br>Please complete the form and click the button below to send your information to the PISCES III team so they can reach out to you with more information. Please do not provide any information about anyone but yourself.<br>To help aid the release of your medical records to ensure we can assess whether you qualify for the study, if you provide your email in the contact form below, we will be emailing you a medical release form. Please complete the form by following the instructions in the email. If you require assistance, the PISCES III study team will be able to help.			
Contact Information	First name*<br>Last name*<br>Address 1*<br>Address 2<br>City*	[ ]<br>[ ]<br>[ ]<br>[ ]<br>[ ]	US state dropdown list<br>US zip code validation (#####)<br>Phone format validation (###-###-####)<br>Email format validation (a@a.a)

	State* [Select ▼]<br>Zip code* [ ]<br>Phone number* [ ]<br>Email address [ ]<br>Alternative Contact Name [ ]	* indicates a required field
Time to Contact	Best time of day to contact you:*<br>○ Morning<br>○ Afternoon<br>○ Evening	* indicates a required field
Privacy Permissions	Your privacy is very important to us. If we are unable to reach you, may we leave a message?*<br>○ Yes<br>○ No<br><br>When you submit your contact information by clicking below, it is forwarded directly to a member of the PISCES III team. By submitting this information, you are not committing to volunteer for this study. You are simply exploring an opportunity to join the study.	* indicates a required field
User Agreement	**User Agreement:** By checking the box below, I understand that the personal information I have provided may be collected, shared, used and/or transferred by the PISCES III team and its staff for the sole purpose of enabling me to be contacted to learn more about the research study, see if I am suitable to take part in this research study, and if appropriate, to assist me in enrolling in this research study. I understand there is no guarantee that I will be contacted by a member of the study team as a result of submitting my personal information. I acknowledge that I may withdraw interest in participating in the research study at any time by speaking with a member of the study team or representative.<br><br>Your information will not be sold or shared with third parties for their own separate use.<br><br>□ By checking this box, I verify that:*<br>– The name and phone numbers I have provided belong to me, are correct, and that I may be interested in participating in this research study.<br>– I am 18 years or older.<br>– I have read and understood the Legal Notice and Privacy Statement.<br><br>[Click Here to Send Information to the PISCES III Study Team]	**Privacy Policy** links to Screen 11<br>**Legal Notice** links to Screen 12<br><br>**Click Here to Send Information to Study Center:** Required fields filled in + Time of day selected + message permission selected + User agreement checked: Continue to Screen 5

**Screen #5: Pre-Qualified Thank You Text** (thankyoupq.aspx)
Thank You<br>Your information has been submitted and a member of the PISCES III team will contact you with more information.<br>If you have questions, please contact the PISCES III team by calling 1-844-707-8336 or emailing PISCES3@duke.edu.<br>Please don't forget to check your email for the medical release form.<br>Thank you for your interest in this research study.<br>Have a great day.

**Screen #6: Not Qualified Contact Information Text** (disqualify.aspx)

<table>
<tr><td colspan="2">

Sorry, You Do Not Qualify

Programming note: the following text in brackets will appear for users who have ***only*** disqualified on Question "Most recent stroke (months)/4" with answer "Less than 3 months ago":
[Due to the timing of your last stroke being less than 3 months ago, we are sorry you are not currently eligible for the PISCES III study.
However, if you decide to provide your contact information, we would like to reach out to you in the near future to see if you're still interested in further pre-screening for the study.
Your privacy is very important to us. This information will only be used to inform you of clinical research studies.]

Programming note: the following text in brackets will appear for users who have disqualified for any other question/combination:
[Based upon the information you have provided, we are sorry you are not eligible for the PISCES III study. With your permission, the information you have provided will be kept on file with Clinical Trial Media so that we may contact you regarding this study or for future studies.
Your privacy is very important to us. This information will only be used to inform you of clinical research studies. It will never be disclosed to third parties except as required by law.]

Do we have your permission to retain your personal information in our files? If you choose "No" we will not ask you for your contact information.
o Yes
o No

(Reveal contact info section below only if 'Yes' is selected)

Please verify your contact information in the form below and then click submit.
</td><td>**Yes:** Reveal contact form as indicated
No: Route to Screen 8</td></tr>
<tr><td>Contact Information</td><td>First name*
Last name*
Address 1*
Address 2
City*
State*   Select ▼
Zip code*</td><td>US state dropdown list
US zip code validation (#####)
Phone format validation (###-###-####)
Email format validation (a@a.a)
* indicates a required field</td></tr>
</table>

	Phone number* [ ]<br>Email address [ ]<br>Alternative Contact Name [ ]	
User Agreement	**User Agreement:** By checking the box below, I understand that the personal information have provided may be collected, shared, used and/or transferred by the PISCES III team and its staff for the sole purpose of enabling me to be contacted to learn more about the research study, see if I am suitable to take part in this research study, and if appropriate, to assist me in enrolling in this research study. I understand there is no guarantee that I will be contacted by a member of the study team as a result of submitting my personal information. I acknowledge that I may withdraw interest in participating in the research study at any time by speaking with a member of the study team or representative.<br><br>Your information will not be sold or shared with third parties for their own separate use.<br><br>□ By checking this box, I verify that:<br>– The name and phone numbers I have provided belong to me, are correct, and that I may be interested in participating in this research study.<br>– I am 18 years or older.<br>– I have read and understood the Legal Notice and Privacy Statement.	**Privacy Policy** links to Screen 11<br>**Legal Notice** links to Screen 12
[Submit]		**Submit:** Required fields filled in + User agreement checked: Route to Screen 8

**Screen #8: DQ/No Interest Thank You Text** (nointerest.aspx)
Thank You<br><br>Thank you for your interest in the PISCES III study.<br><br>If you are interested in learning more about current and future clinical studies coordinated by Duke, please visit the Duke Neuroscience volunteer registry by clicking here *(programming note, click here links to: https://duke.qualtrics.com/jfe/form/SV_0ugScZmU8LZHK9D)* or by calling 1-833-383-4870.<br><br>Have a great day.

**Screen #9: Frequently Asked Questions (FAQ.aspx) – NOT APPLICABLE**

**Screen #10: About Clinical Trials** (about.aspx)
About Research Studies<br><br>What is a research study?<br>Why are research studies important?<br>Where can people find out about research studies?<br>Why should I consider participating in a research study?<br>Where are research studies conducted?<br>What is "informed consent"?<br><br>What is a research study?<br>A research study (also known as a clinical trial or investigation) is a medical study that is designed to answer questions about the safety and effectiveness of potential new drugs. Research studies must be performed before a potential new drug can be approved for use in patients.<br><br>Why are research studies important?<br>Research studies are used to test potential medications before they can become available to the general public. The testing that takes place during such research studies provides information regarding the safety and effectiveness of the potential medication.<br><br>Where can people find out about research studies?<br>One way to find information about clinical trials is by searching this website: www.ClinicalTrials.gov. ClinicalTrials.gov is an interactive online database, managed by the National Library of Medicine. It provides information about both federally and privately supported clinical research. ClinicalTrials.gov is updated regularly and offers information on each trial's purpose, who is eligible to participate, locations, and phone numbers to call for more information.<br><br>Why should I consider participating in a research study?<br>For those who are eligible, taking part in research studies offers several benefits:<br>• Getting actively involved in their own health care<br>• Having access to potentially new research treatments<br>• Having access to expert medical care for the condition being studied, since investigators are often specialists in the disease area being studied

- Helping others by contributing to medical research

It is important to test drugs and medical products in the people they are meant to help. It is also important to conduct research in a variety of people because different people may respond differently to treatments.

For each research study, researchers develop eligibility criteria, such as age, gender, previous treatment history, and other medical conditions. Not everyone who applies for a research study will be accepted. Volunteers may be excluded based on the eligibility criteria and/or the number of participants needed by the researchers.

Where are research studies conducted?

Research studies can be sponsored by an organization such as a pharmaceutical company, a federal agency such as the Veterans Administration, or an individual, such as a physician or health care provider. The sponsor determines the location(s) of the trials, which are usually conducted at universities, medical centers, clinics, doctor's offices, and/or at hospitals.

What is “informed consent”?

The government requires researchers to give prospective participants complete and accurate information about what will happen during the study. Participants must sign an "informed consent" form before joining the study, indicating they understand that the study is research, and that they can leave the research study at any time. This informed consent helps ensure that a prospective research study participant understands what’s involved.

**Screen #11: Privacy Policy** (privacypolicy.aspx)
**CliniCallRN™ (a dba of Clinical Trial Media, Inc.)**<br>**Privacy Policy**<br>**Effective Date: 6/18/2018**<br><br>**INTRODUCTION**<br>CliniCallRN™ is committed to respecting and protecting your privacy.<br>This privacy policy sets out how we look after your personal data, how we will use your personal data, and tells you about your privacy rights and how the law protects you.<br>This privacy policy sets out our approach to protecting personal data on a worldwide basis and we recognize that different jurisdictions and legal systems will apply:<br>(i) In the United States, the Federal Trade Commission has jurisdiction over our compliance regarding personal data. If you do not agree to the terms of this privacy policy, you should not access or use CliniCallRN™.<br>(ii) In the rest of the world, different legal rules apply and, in particular, we will be using and protecting personal data in a way which is in accordance with the rules operating in the

European Economic Area (“EEA”) which has adopted the General Data Protection Regulation (“GDPR”). In the EEA, the relevant national supervisory authority will have jurisdiction over our compliance in any specific EEA country. If you do not agree to the terms of this privacy policy, please do not access or use CliniCallRN™.

TABLE OF CONTENTS

1. PURPOSE
2. THE DATA WE COLLECT ABOUT YOU
3. HOW YOUR PERSONAL DATA IS COLLECTED
4. HOW WE USE YOUR PERSONAL DATA
5. DISCLOSURES OF YOUR PERSONAL DATA
6. INTERNATIONAL TRANSFERS
7. DATA SECURITY
8. DATA RETENTION
9. YOUR DATA PRIVACY RIGHTS UNDER GDPR
10. DEFINITIONS

1. PURPOSE

This privacy policy describes how CliniCallRN™ collects, uses, processes and protects your personal data and informs the choices available to you regarding how you can choose and manage your personal data.

It is important that you read this privacy policy together with any other privacy policy or fair processing notice we may provide on specific occasions when we are collecting or processing personal data about you so that you are fully aware of how and why we are using your data. This privacy policy supplements the other notices and is not intended to override them.

1.1 CONTROLLER

This privacy policy is issued on behalf of CliniCallRN™ so when “we”, “us” or “our” is mentioned in this privacy policy, we are responsible for processing your data.

We have appointed a data protection officer who is responsible for dealing with questions in relation to this privacy policy. If you have any questions about this privacy policy, including any requests to exercise your legal rights, please contact the data protection officer using the details set out below.

1.2 CONTACT DETAILS

Our contact details are:
Full name of legal entity: Clinical Trial Media, Inc.
Name or title of data protection officer: Kenneth S. King
Email address: privacy@clinicallrn.com
Telephone number: 516-366-5665

Postal address: 308 Harper Drive, Suite 105, Moorestown, NJ 08057, USA
URL: http://www.clinicallrn.com/GDPRRequestForm.php

1.3 COMPLAINTS (GDPR for EEA INDIVIDUALS)

In the EEA, you have the right to make a complaint at any time to the relevant national supervisory authority. To find more about this right and to locate the appropriate Data Privacy Authority, go to the European Commission website (https://ec.europa.eu/info/policies/justice-and-fundamental-rights_en) if in the UK, go to the Information Commissioner's Office ("ICO") website (www.ico.org.uk). We would, however, appreciate the chance to deal with your concerns before you approach one of the national supervisory authorities, so please contact us in the first instance.

1.4 COMPLAINTS (EU-US and Swiss-US Privacy Shield)

In compliance with the Privacy Shield Principles, CliniCallRN™ commits to resolve complaints about your privacy and our collection or use of your personal information transferred to the United States pursuant to Privacy Shield. European Union and Swiss individuals with Privacy Shield inquiries or complaints should first contact us by email at privacy@clinicallrn.com.

CliniCallRN™ has further committed to refer unresolved privacy complaints under the Privacy Shield Principles to an independent dispute resolution mechanism, the BBB EU PRIVACY SHIELD, operated by the Council of Better Business Bureaus. If you do not receive timely acknowledgment of your complaint, or if your complaint is not satisfactorily addressed, please visit www.bbb.org/EU-privacy-shield/for-eu-consumers for more information and to file a complaint. This service is provided free of charge to you.

If your Privacy Shield complaint cannot be resolved through the above channels, under certain conditions, you may invoke binding arbitration for some residual claims not resolved by other redress mechanisms. See Privacy Shield Annex 1 at https://www.privacyshield.gov/article?id=ANNEX-I-introduction

CliniCallRN™ complies with the US-EU and Swiss-US Privacy Shield Principles, we commit to resolving complaints about your privacy and our collection and/or use of your personal data. Accordingly, any individuals with enquiries or complaints regarding our use of your personal data or this privacy policy should contact us at the details given in the Contact Details section of this notice. In addition, CliniCallRN™ has further committed to refer unresolved privacy complaints to an independent dispute resolution mechanism, the BBB EU Privacy Shield, operated by the Council of Better Business Bureaus (please visit https://www.bbb.org/EU-privacy-shield/file-a-complaint/ for more information and to file a complaint visit https://www.auto.bbb.org/eu-privacy-shield-complaint-form/).

1.5 CHANGES TO THE PRIVACY POLICY AND YOUR DUTY TO INFORM US OF CHANGES IN YOUR PERSONAL DATA

We reserve the right to amend this privacy policy and will notify you by updating this notice, so please check it from time to time, especially if you have ongoing dealings with us. It is important that the personal data we hold about you is accurate and current. Please keep us informed if your personal data changes during your relationship with us.

1.6 THIRD-PARTY LINKS

This website may include links to third-party websites, plug-ins and applications. Clicking on those links or enabling those connections may allow third parties to collect or share data about you. We do not control these third-party websites and are not responsible for their privacy statements. When you leave our website, we encourage you to read the privacy policy of every website you visit

2. THE DATA WE COLLECT ABOUT YOU

Personal data, or personal information, means any information about an individual from which that person can be identified. It does not include data where the identity has been removed (anonymized data).

We may collect, use, store and transfer different kinds of personal data about you which we have grouped together as follows:

- **Identity Data** includes first name, maiden name, last name, username or similar identifier, date of birth, and gender.
- **Contact Data** includes billing address, delivery address, email address and telephone numbers.
- **Financial Data** includes banking details of clients, suppliers, and agents for the making of payments by us and to us in relation to the services we provide.
- **Transaction Data** includes details of products and services you have received or purchased from us and/or affiliates.
- **Technical Data** includes internet protocol ("IP") address, your login data, browser type and version, time zone setting and location, browser plug-in types and versions, operating system and platform, and other technology on the devices you use to access this website.
- **Profile Data** includes your username and password, purchases or orders made by you, your interests, preferences, feedback, and survey/questionnaire responses.
- **Usage Data** includes information about how you use our website, products, and services.
- **Marketing and Communications Data** includes your preferences in receiving marketing from us and/or affiliates.
- **Health Data** includes information in relation to any aspect of your health and/or consequences of taking part in any clinical trials organized by our clients.

We may also collect, use and share **Aggregated Data** such as general statistical or demographic data for any purpose. Aggregated Data may be derived from your personal data but is not considered personal data in law as this data does **not** directly or indirectly reveal your identity. For example, we may aggregate your Usage Data to calculate the percentage of users accessing a specific website feature. However, if we combine or connect Aggregated Data with your personal data so that it can directly or indirectly identify you, we treat the combined data as personal data which will be used in accordance with this privacy policy.

Apart from Health Data and industry-wide or governmental survey(s)/questionnaire(s) where we are obliged to take part, we do not normally collect any **Special Categories of Personal Data** about you (this includes details about your race or ethnicity, religious or philosophical beliefs, sex life, sexual orientation, political opinions, and trade union membership.)

2.1 IF YOU FAIL TO PROVIDE PERSONAL DATA

Where we need to collect personal data by law, or under the terms of a contract we have with you and you fail to provide that data when requested, we may not be able to perform the contract we have or are trying to enter into with you (for example, to provide you with our services). In this case, we may have to cancel a product or service you have with us but we will notify you if this is the case at the time.

3. HOW YOUR PERSONAL DATA IS COLLECTED

We use different methods to collect data from and about you including through:

- **Direct interactions.** You may give us your personal data by filling in forms or by corresponding with us by mail, phone, and email, or otherwise. This includes personal data you provide when you:
    - apply online or otherwise for our services or products;
    - contract to receive our services; or
    - request marketing material to be sent to you.
- **Automated technologies or interactions.** As you interact with our website, we may automatically collect Technical Data about your equipment, browsing actions and patterns. We collect this personal data by using cookies, log files, and other similar technologies. We may also receive Technical Data about you if you visit other websites employing our cookies. This aggregate data gives a "macro-view" of the visitor traffic pattern and insight to what sections of the website users visits most. We use this information to determine what kind of technology is available on the visitors' computers so it can better serve them by utilizing more advanced technologies (e.g., Macromedia Flash). None of this information is linked to any Personal Information.
    - We passively collect and log the following information from visitors to our site such as:
        - Browser type
        - IP Address
        - Domain Name
        - Access Time
        - Operating System
- **Third parties or publicly available sources.** We may receive personal data about you from various third parties and public sources as set out below:
    - We may receive **Technical Data** from the following parties:

      (a)analytics providers such as Google;

      (b)advertising networks;

      (c)search information providers;

      (d)portals.

    - **Contact and Transaction Data** from providers of technical, payment and delivery services.
    - **Identity and Contact Data** from data brokers or aggregators.

4. HOW WE USE YOUR PERSONAL DATA

We will only use your personal data when the law allows us to. Most commonly, we will use your personal data in the following circumstances:

- Where we need to perform the contract we are about to enter into or have entered into with you, or to perform other legal obligations.
- Where it is necessary for our legitimate interests (or those of a third party) and your interests and fundamental rights do not override those interests (this applies in the EEA).
- Where we need to comply with a legal or regulatory obligation.

In the EEA, in relation to sending direct marketing communications to you via email or text message, we will only do so where (i) we have your express consent or (ii) you are an existing client. You have the right to withdraw consent to marketing at any time by contacting us.

4.1 PURPOSES FOR WHICH WE WILL USE YOUR PERSONAL DATA

We have set out below, in table format, a description of the ways we plan to use your personal data, and which of the legal basis we rely on to do so. We have also identified what our legitimate interests are, where appropriate. This table has been partly drawn up as a result of the recent GDPR rules being brought into force in the EEA but is also relevant to our use of personal data in other parts of the world.

Note that we may process your personal data for more than one lawful ground depending on the specific purpose for which we are using your data. Please contact us if you need details about the specific legal ground we are relying on to process your personal data where more than one ground has been set out in the table below.

**Purpose/Activity**	**Type of data**	**Lawful basis for processing including basis of legitimate interest**
To register you as a new customer	(a) Identity<br>(b) Contact	Performance of a contract with you

To process and deliver services and/or perform contractual obligations for you, including collecting and recovering money owed to us	(a) Identity<br><br>(b) Contact<br><br>(c) Financial<br><br>(d) Transaction<br><br>(e) Marketing and Communications	(a) Performance of a contract with you<br><br>(b) Necessary for our legitimate interests (to recover funds due to us)
To manage our relationship with you which will include:<br><br>(a) Notifying you about changes to our terms or privacy policy<br><br>(b) Asking you to leave a review or take a survey/questionnaire	(a) Identity<br><br>(b) Contact<br><br>(c) Profile<br><br>(d) Marketing and Communications	(a) Performance of a contract with you<br><br>(b) Necessary to comply with a legal obligation<br><br>(c) Necessary for our legitimate interests (to keep our records updated and to study how customers use our products/services)
To enable you to complete a survey/questionnaire	(a) Identity<br><br>(b) Contact<br><br>(c) Profile<br><br>(d) Usage<br><br>(e) Marketing and Communications	(a) Performance of a contract with you<br><br>(b) Necessary for our legitimate interests (to study how customers use our products/services, to develop them and grow our business)
To consider whether you are eligible/suitable for taking part in a specific clinical trial, related clinical investigation, or clinical support program carried our clients	(a) Identity<br><br>(b) Contact<br><br>(c) Health	(a) Necessary for our legitimate interests to develop our products/services

		(b) Necessary in order to comply with contractual obligations with our end-clients
To administer and protect our business and this website (including troubleshooting, data analysis, testing, system maintenance, support, reporting, and hosting of data)	(a) Identity<br><br>(b) Contact<br><br>(c) Technical	(a) Necessary for our legitimate interests (for running our business, provision of administration and IT services, network security, to prevent fraud and in the context of a business reorganization or group restructuring exercise)<br><br>(b) Necessary to comply with a legal obligation<br><br>(c) Necessary to resolve disputes
To deliver relevant website content and advertisements to you and measure or understand the effectiveness of the advertising we serve to you	(a) Identity<br><br>(b) Contact<br><br>(c) Profile<br><br>(d) Usage<br><br>(e) Marketing and Communications<br><br>(f) Technical	Necessary for our legitimate interests (to study how customers use our products/services, to develop them, to grow our business and to inform our marketing strategy)
To use data analytics to improve our website, products/services, marketing, customer relationships and experiences; provide audit record for consent	(a) Technical<br><br>(b) Usage	Necessary for our legitimate interests (to define types of customers for our products and services, to keep our website updated and relevant, to develop our business and to inform our marketing strategy)
To make suggestions and recommendations to you	(a) Identity	Necessary for our legitimate interests (to develop our

about goods or services that may be of interest to you	(b) Contact<br>(c) Technical<br>(d) Usage<br>(e) Profile	products/services and grow our business)

4.2 DISCLOSING INFORMATION TO THIRD-PARTIES

We will get your express consent before we share your personal data with any company outside CliniCallRN™.

We do not sell your personal data to any third party. Our use and disclosure of Personal Identifiable Health Information ("PIHI") is limited to the minimum amount of personal data needed to accomplish the intended purpose of the specific clinical investigation or clinical trial and is used in relation to pre-screening activities for such clinical research projects. This includes using study questionnaires that only ask health and medical related questions that are directly associated with the relevant clinical research project as specified in approved protocols.

PIHI will generally not be used by us or disclosed by us to any third parties unless we have clear consent from you to do so.

Exceptionally, PIHI may be disclosed by us where we are required to do so by a relevant law or regulation. In particular, this includes, but is not limited to, situations where we are required to disclose such PIHI in relation to requests by public authorities to meet national security or law enforcement requirements. This will include use and/or disclosure in order to:

- prevent or control disease, injury or disability;
- report disease, injury or disability;
- assist public health surveillance, investigations or interventions;
- report child abuse or neglect or domestic violence;
- avert a serious threat to individual(s) or public health or safety;
- to coroners and/or medical examiners or for tissue donation;
- in response to legal proceedings and relevant court orders or subpoenas;

- for specialized government functions and worker's compensation;
- by workforce members who are whistle-blowers or victims of a criminal act;
- when we believe in good faith that disclosure is necessary to protect our rights or to protect your safety, the safety of others or investigate fraud.

4.3 OPTING OUT

You can ask us or third parties to stop sending you information/reminder messages at any time by contacting us.

Where you opt out of receiving these information/reminder messages, this will not apply to personal data provided to us as a result of a product/service purchase, warranty registration, product/service experience or other transactions.

4.4 COOKIES

We only use cookies to record user-specific information on what pages users' access or visit, record past activity, and session management and personalization. Use of cookies allows a better user experience when visitors return to the website.

You can set your browser to refuse all or some browser cookies, or to alert you when websites set or access cookies. If you disable or refuse cookies, please note that some parts of the website may become inaccessible or not function properly.

4.4.1 Cookie Control. CliniCallRN™'s interactive cookie statement clearly states how the user's behavior is tracked and offers easy-to-use controls for granting and revoking consent. The user has the control to prevent cookies from being placed on their computer until consent via an affirmative act.

4.5 CHANGE OF PURPOSE

We will only use your personal data for the purposes for which we collected it, unless we reasonably consider that we need to use it for another reason and that reason is compatible with the original purpose.

If we need to use your personal data for an unrelated purpose, we will notify you and we will explain the legal basis which allows us to do so.

Please note that we may process your personal data without your knowledge or consent, in compliance with the above rules, where this is required or permitted by law.

4.6 USE OF HEALTH DATA IN THE UNITED STATES

The Health Insurance Portability and Accountability Act of 1996 ("HIPAA") and subsequent regulations published by the Department of Health and Human Services ("DHHS") impose restrictions on other organizations (Covered Entities) which may be covered under HIPAA with respect to your relationship with CliniCallRN™. CliniCallRN™ may, in providing subject recruiting call center services for one of these organizations, be required to comply with certain aspects of HIPAA in their conduct of human subject research activities.

Although CliniCallRN™ is not a Covered Entity as defined in the HIPAA privacy regulations, our policies and procedures, which govern the privacy rights of research participants included in this privacy policy, are compatible with those required by HIPAA for Covered Entities and will become standard for research activities involving PIHI.

All PIHI data collected by CliniCallRN™ in connection with subject recruiting for a clinical research study is captured electronically and transmitted through a secure network connection to a secure database. CliniCallRN™'s data security policies are consistent with Good Clinical Practices, HIPAA and GDPR standards. CliniCallRN™ maintains separate Security Policies for Physical Security, Network Security and Application Security.

5. DISCLOSURES OF YOUR PERSONAL DATA

We may have to share your personal data with the parties set out below for the purposes set out in the table in section 4.1 above.

- Third party sub-contractors who provide services for us and/or help to provide services to you. In the event that we use sub-contractors who have access to your personal data, we ensure that there are strict contractual terms in place to ensure that they only process personal data to the extent that we instruct them to do so in writing and there are suitably worded confidentiality and data protection clauses in all such contracts.
- Third parties to whom we may choose to sell, transfer, or merge parts of our business or our assets. Alternatively, we may seek to acquire other businesses or merge with them. If a change of control arises in relation to our business, then the new owners may use your personal data in the same way as set out in this privacy policy.

We require all third parties to respect the security of your personal data and to treat it in accordance with the law. We do not allow our third-party service providers to use your personal data for their own purposes and only permit them to process your personal data for specified purposes and in accordance with our instructions.

6. INTERNATIONAL TRANSFERS

Some personal data may be held on servers in the US. This will involve transferring your data outside the European Economic Area ("EEA"). In addition, we use third parties who have IT servers located in the United States which hold your personal data.

Whenever we transfer and/or process your personal data outside of the EEA, we ensure a similar degree of protection is afforded to it by ensuring at least one of the following safeguards is implemented:

- where we use certain service providers, we may use specific contracts approved by the European Commission which give personal data the same protection it has in Europe.
  - where we use providers based in the US, we may transfer data to them if they are part of the Privacy Shield which requires them to provide similar protection to that afforded in the EEA to personal data shared between the Europe and the US.

6.1 PRIVACY SHIELD FRAMEWORKS

Clinical Trial Media, Inc. dba CliniCallRN™ complies with the EU-US Privacy Shield Framework and the Swiss-US Privacy Shield Framework as set forth by the US Department of Commerce regarding the collection, use, and retention of personal information from European Union member countries and Switzerland transferred to the United States pursuant to Privacy Shield.

CliniCallRN™ has certified that it adheres to the Privacy Shield Principles with respect to such data. If there is any conflict between the policies in this privacy policy and data subject rights under the Privacy Shield Principles, the Privacy Shield Principles shall govern. To learn more about the Privacy Shield program, and to view our certification page, please visit https://www.privacyshield.gov/

With respect to personal data received or transferred pursuant to the Privacy Shield Frameworks, CliniCallRN™ is subject to the regulatory enforcement powers of the U.S. Federal Trade Commission.

Pursuant to the Privacy Shield Frameworks, EU and Swiss individuals have the right to obtain our confirmation of whether we maintain personal information relating to you in the United States. Upon request, we will provide you with access to the personal information that we hold about you. You may also may correct, amend, or delete the personal information we hold about you. An individual who seeks access, or who seeks to correct, amend, or delete inaccurate data transferred to the United States under Privacy Shield, should direct their query to privacy@clinicallrn.com. If requested to remove data, we will respond within a reasonable timeframe.

We will provide an individual opt-out or opt-in choice before we share your data with third parties other than our agents, or before we use it for a purpose other than which it was originally collected or subsequently authorized. To request to limit the use and disclosure of your personal information, please submit a written request to privacy@clinicallrn.com.
In certain situations, we may be required to disclose personal data in response to lawful requests by public authorities, including to meet national security or law enforcement requirements.

CliniCallRN™'s accountability for personal data that it receives in the United States under the Privacy Shield and subsequently transfers to a third party is described in the Privacy Shield Principles. In particular, CliniCallRN™ remains responsible and liable under the Privacy Shield Principles if third-party agents that it engages to process the personal data on its behalf do so in a manner inconsistent with the Principles, unless CliniCallRN™ proves that it is not responsible for the event giving rise to the damage.

In compliance with the Privacy Shield Principles, CliniCallRN™ commits to resolve complaints about your privacy and our collection or use of your personal information transferred to the United States pursuant to Privacy Shield. European Union and Swiss individuals with Privacy Shield inquiries or complaints should first contact us by email at privacy@clinicallrn.com.

CliniCallRN™ has further committed to refer unresolved privacy complaints under the Privacy Shield Principles to an independent dispute resolution mechanism, the BBB EU PRIVACY SHIELD, operated by the Council of Better Business Bureaus. If you do not receive timely acknowledgment of your complaint, or if your complaint is not satisfactorily addressed, please visit www.bbb.org/EU-privacy-shield/for-eu-consumers for more information and to file a complaint. This service is provided free of charge to you.

If your Privacy Shield complaint cannot be resolved through the above channels, under certain conditions, you may invoke binding arbitration for some residual claims not resolved by other redress mechanisms. See Privacy Shield Annex 1 at https://www.privacyshield.gov/article?id=ANNEX-I-introduction

7. DATA SECURITY

We and our third party hosting partners have put in place appropriate security measures to prevent your personal data from being accidentally lost, used or accessed in an unauthorized way, altered or disclosed. In addition, we limit access to your personal data to those employees, agents, contractors and other third parties who have a business need to know. They will only process your personal data on our instructions and they are subject to a duty of confidentiality.

We have put in place procedures to deal with any suspected personal data breach and will notify you and any applicable regulator of a breach where we are legally required to do so.

8. DATA RETENTION

We will only retain your personal data for as long as necessary to fulfill the purposes we collected it for, including for the purposes of satisfying any legal, accounting, or reporting requirements.

To determine the appropriate retention period for personal data, we consider the amount, nature, and sensitivity of the personal data, the potential risk of harm from unauthorized use or disclosure of your personal data, the purposes for which we process your personal data and whether we can achieve those purposes through other means, and the applicable legal requirements.

In some circumstances in the EEA you can ask us to delete your data: see the section below entitled "Your Data Privacy Rights in the EEA" for further information.

We may also anonymize your personal data (so that it can no longer be associated with you) for research or statistical purposes in which case we may use this information indefinitely without further notice to you.

9. YOUR DATA PRIVACY RIGHTS UNDER GDPR

Under certain circumstances in the EEA, you have the following rights under data protection laws in relation to your personal data:

Request access to your personal data (commonly known as a "data subject access request"). This enables you to receive a copy of the personal data we hold about you and to check that we are lawfully processing it.

Request correction of the personal data that we hold about you. This enables you to have any incomplete or inaccurate data we hold about you corrected, though we may need to verify the accuracy of the new data you provide to us.

Request erasure of your personal data. This enables you to ask us to delete or remove personal data where there is no good reason for us continuing to process it. You also have the right to ask us to delete or remove your personal data where you have successfully exercised your right to object to processing (see below), where we may have processed your information unlawfully or where we are required to erase your personal data to comply with local law. Note, however, that we may not always be able to comply with your request of erasure for specific legal reasons which will be notified to you, if applicable, at the time of your request.

Object to processing of your personal data where we are relying on a legitimate interest (or those of a third party) and there is something about your particular situation which makes you want to object to processing on this ground as you feel it impacts on your fundamental rights and freedoms. You also have the right to object where we are processing your personal data for direct marketing purposes. In some cases, we may demonstrate that we have compelling legitimate grounds to process your information which override your rights and freedoms.

Request restriction of processing of your personal data. This enables you to ask us to suspend the processing of your personal data in the following scenarios: (a) if you want us to establish the data's accuracy; (b) where our use of the data is unlawful but you do not want us to erase it; (c) where you need us to hold the data even if we no longer require it as you need it to establish, exercise or defend legal claims; or (d) you have objected to our use of your data but we need to verify whether we have overriding legitimate grounds to use it.

Request the transfer of your personal data to you or to a third party. We will provide to you, or a third party you have chosen, your personal data in a structured, commonly used, machine-readable format. Note that this right only applies to automated information which you initially provided consent for us to use or where we used the information to perform a contract with you.

Withdraw consent at any time where we are relying on consent to process your personal data. However, this will not affect the lawfulness of any processing carried out before you withdraw your consent. If you withdraw your consent, we may not be able to provide certain products or services to you. We will advise you if this is the case at the time you withdraw your consent.

If you wish to exercise any of the rights set out above, please contact us.

In the EEA, you have the right to make a complaint at any time to the relevant national supervisory authority. For example, in the UK this would be the Information Commissioner's Office ("ICO"), the UK supervisory authority for data protection issues (www.ico.org.uk). We would, however, appreciate the chance to deal with your concerns before you approach one of the national supervisory authorities so please contact us in the first instance.

A list of Supervisory Authorities is available here: http://ec.europa.eu/justice/data-protection/bodies/authorities/index_en.htm.

9.1 NO FEE USUALLY REQUIRED

You will not have to pay a fee to access your personal data or to exercise any of the other rights.

9.2 WHAT WE MAY NEED FROM YOU

We may need to request specific information from you to help us confirm your identity and ensure your right to access your personal data (or to exercise any of your other rights). This is a security measure to ensure that personal data is not disclosed to any person who has no right to receive it. We may also contact you to ask you for further information in relation to your request to speed up our response.

9.3 TIME LIMIT TO RESPOND

We try to respond to all legitimate requests within 30 business days. Occasionally it may take us longer than 30 business days if your request is particularly complex or you have made a number of requests. In this case, we will notify you and keep you updated.

10. DEFINITIONS

EEA

Legitimate Interest means, in the EEA, the interest of our business in conducting and managing our business to enable us to give you the best service/product and the best and most secure experience. We make sure we consider and balance any potential impact on you (both positive and negative) and your rights before we process your personal data for our legitimate interests. We do not use your personal data for activities where our interests are overridden by the impact on you (unless we have your consent or are otherwise required or permitted to by law). You can obtain further information about how we assess our legitimate interests against any potential impact on you in respect of specific activities by contacting us.

Performance of Contract means processing your data where it is necessary for the performance of a contract to which you are a party or to take steps at your request before entering into such a contract.

Comply with a legal or regulatory obligation means processing your personal data where it is necessary for compliance with a legal or regulatory obligation that we are subject to.

GDPR is the European Union General Data Protection Regulation.

UNITED STATES

Covered Entity means an institution, organization or other entity that is subject to the rules of the Health Insurance Portability and Accountability Act of 1996 ("HIPAA"). Covered Entities include: (i) a health plan, (ii) a healthcare clearinghouse and, (iii) a healthcare provider who transmits any personal identifiable health information in electronic form in connection with a transaction covered by HIPAA.

Personal Identifiable Health Information ("PIHI") means any information including demographic information collected from an individual that:

(i) relates to (a) the past, present or future physical or mental health or condition of an individual; (b) the provision of healthcare to an individual; or (c) the past, present or future payment for the provision of healthcare to the individual; and

(ii) identifies the individual or there is a reasonable basis to believe it can be used to identify the individual; and

(iii) PIHI does not include education records or medical records covered by the Family Education Rights and Privacy Act or employment records held by CliniCallRN™ in its role as an employer.

THIRD PARTIES

- Service providers acting as processors and who provide services to us.
- Professional advisers acting as processors or joint controllers including lawyers, bankers, auditors and insurers who provide consultancy, banking, legal, insurance and accounting services to us.
- Regulators and other state authorities acting as processors or joint controllers in any jurisdiction in which we are operating and who require reporting of processing activities in certain circumstances.

**Screen #12: Legal Notice** (legalnotice.aspx)

Legal Notice

Acceptance of Agreement. Your access to and use of www.PISCES3.org, website (the "Site"), which is maintained by Clinical Trial Media, Inc. (www.clinicaltrialmedia.com) is subject to the following terms and conditions and all applicable laws. By accessing and browsing the Site, you accept and agree to, without limitation or qualification, the terms and conditions and acknowledge that any other agreements between you and Clinical Trial Media are superseded and of no force or effect with respect to your access and use of the Site.

Scope of Use. Clinical Trial Media maintains the Site for your personal information, education, and communication. Please feel free to browse the Site. You may download material displayed on the Site for non-commercial, personal use only, provided you maintain all copyright and other proprietary notices on the materials. You may not, however, distribute, modify, transmit, reuse, repost or use the content of the Site for commercial purposes, including the text and images, without Clinical Trial Media's written permission. You understand that Clinical Trial Media makes no representation that the information in the Site is appropriate or available for use in locations outside of the United States, and access to the Site from territories where the content of the Site may be illegal or inappropriate is prohibited. Those who choose to access the Site from other locations do so on their own initiative and are responsible for compliance with applicable local laws.

No Medical Advice. The Site does not provide medical advice. With limited exception, any products discussed on the Site are available only by prescription from a licensed health care professional. The Site is not engaged in rendering medical or similar professional services or advice, and the information provided on the Site is not intended to replace medical advice offered by a health care provider. If you desire or need such services or advice, you should promptly consult a physician or professional health care provider.

Product Information. The product information provided on the Site is intended only for residents in the United States. The Site and its links may, however, contain information about products that may or may not be available in any particular country, territory or region of the world (including the United States), may be available under different trademarks in different countries and, where applicable, may be approved or cleared by a government regulatory body for sale or use with different indications and restrictions in different countries. You should not construe anything on the Site as a promotion or solicitation for any product or service or for the use of any product or service that is not authorized by the laws and regulations of the country where you are located, including the United States. For specific information about particular products, you should refer to the full prescribing information as approved by the relevant governmental authority of the country where you are located. The prescribing information provided here is based on United States labeling and may not be appropriate outside of the United States.

Copyright Protection. You should assume that everything you see or read on the Site is copyrighted, unless otherwise noted, and may not be used except as provided in these terms and conditions or in the text on the Site without the written permission of the Site. The Site neither warrants nor represents that your use of materials displayed on the Site will not infringe rights of third parties not owned by or affiliated with the Site.

Trademarks. The trademarks, trade names, logos and service marks (collectively the "Trademarks") displayed on the Site are registered and unregistered Trademarks of Clinical Trial Media and others. Nothing contained on the Site should be construed as granting, by implication, estoppel or otherwise, any license or right to use any Trademark displayed on the Site without the written permission of Clinical Trial Media or any third party that may own the Trademarks displayed on the Site. Your use of the Trademarks displayed on the Site, or any other content on the Site, except as provided in these terms and conditions, is strictly prohibited. You are also advised that Clinical Trial Media will aggressively enforce its intellectual property rights to the fullest extent of the law, including the seeking of criminal prosecution.

Exclusion of Warranty. While Clinical Trial Media uses reasonable efforts to include accurate and up-to-date information in the Site, Clinical Trial media makes no warranties or representations as to its accuracy. Clinical Trial media assumes no liability or responsibility for any errors or omissions in the content of the Site. Without limiting the foregoing, everything on the Site is provided to you "AS IS" WITHOUT WARRANTY OF ANY KIND, EITHER EXPRESSED OR IMPLIED, INCLUDING, BUT NOT LIMITED TO, THE IMPLIED WARRANTIES OF MERCHANTABILITY, FITNESS FOR A PARTICULAR PURPOSE OR NON-INFRINGEMENT. Please note that some jurisdictions may not allow the exclusion of implied warranties, so some of the above exclusions may not apply to you. Check your local laws for any restrictions or limitations regarding the exclusion of implied warranties.

Limitation of Liability. Your use of and browsing in the Site are at your risk. Neither the Site nor any other party involved in creating, producing or delivering the Site is liable for any direct, incidental, consequential, indirect or punitive damages arising out of your access to, or use of, the Site. The Site and/or Clinical Trial Media assume no responsibility, and shall not be liable for, any damages to, or viruses that may infect, your computer equipment or other property on account of your access to, use of, or browsing in the Site or your downloading of any materials, data, text or images from the Site.

Prohibited Transmissions. Certain areas of the Site may enable you to access online forums and to submit e-mails, or otherwise provide feedback to the Site. Clinical Trial Media does not endorse, and specifically disclaims, any responsibility or liability for any content in these forays. You may not post or transmit any unlawful, threatening, libelous, defamatory, obscene, indecent, inflammatory, pornographic or profane material or any material that could constitute or encourage conduct that would be considered a criminal offense, give rise to civil liability or otherwise violate any law. In addition, you are prohibited from posting or transmitting any information which (a) infringes the rights of others or violates their privacy or publicity rights, (b) is protected by copyright, trademark or other proprietary right, unless with the express written permission of the owner of such right, (c) contains a virus, bug or other harmful item, or (d) is used to unlawfully collude against another person in restraint of trade or competition. You shall be solely liable for any damages resulting from any infringement of copyright, trademark, or other proprietary right, or any other harm resulting from your use of the Site.

Use Submissions. Any "personally identifiable information" in electronic communications to the Site is governed by the Site's Privacy Policy. On the other hand, any communication or material you transmit to the Site by electronic mail or otherwise, including any data, questions, comments, suggestions or the like is, and will be treated as, non-confidential and non-proprietary. You understand that anything you transmit or post may be used by Clinical Trial Media or its affiliates for any purpose, including but not limited to, reproduction, disclosure, transmission, publication, broadcast, and posting. Furthermore, Clinical Trial Media is free to use any ideas, concepts, know-how, processes, or techniques contained in any communication you send to the Site for any purpose whatsoever including, but not limited to, developing, manufacturing, marketing, and selling products and service using such ideas, concepts, know-how, processes, or techniques.

Links to Other Sites. Clinical Trial Media has not reviewed all of the sites linked to the Site and is not responsible for the content of any off-site pages or any other sites linked to the Site. Your linking to any other off-site pages or other sites is at your own risk. Once you link to another site, please be sure to review the legal notice and privacy policy of the new site. It may be different from those governing the Site.

Modification of Agreement. Clinical Trial Media may, at any time, revise the terms and conditions of this agreement governing use of the Site. If any terms and conditions contained in this Legal Notice are changed, the amended notice will be posted on the Site. You are bound by any such revisions and should, therefore, periodically review these terms and conditions.

Sponsor/Client:	ReNeuron	Document:	ReNeuron_PiscesIII_Stroke_Call Center Script_V 2.3_9.26.19_US English_CLEAN.docx
Protocol:	RN01-CP-0003 Pisces III Stroke	Version:	V2.3
		Status:	Protocol Amendment

**Category**<br>*[no IRB review]*	**Programming Label**<br>*[no IRB review]*	**Content for IRB Review**<br>[Text in blue is spoken to callers]	**Programming Notes**<br>*[no IRB review]*
Introduction	Block 1 (intro)	**INBOUND CALL**<br>Thank you for calling our clinical research information center. My name is [Agent Name]. Are you calling about the PISCES III research study?<br><br>**OUTBOUND (if patient leaves a voicemail)**<br>Hello, may I speak with [name]?<br><br>Hello, my name is [Agent Name]. I am a nurse (or other applicable credential) returning a call about the PISCES III research study. We are currently recruiting volunteers to participate in this research study. Are you interested in learning more?<br><br>**OUTBOUND (if voicemail is reached)**<br>This is [Agent Name] from the clinical research information center with a message for [lead name] regarding a new clinical research study. If you are interested in learning more about the study and seeing if you pre-qualify, please call back at [call center callback number] between the hours 8am and 8pm Eastern time and any of the nurses who answer can assist you.	Inbound Dispositions:<br>**Study:** Continue<br>**Hang-up:** Route to end of script<br>**Wrong Number:** Route to end of script<br>**Other:** Route to end of script<br><br>Outbound – Live Answer Dispositions:<br>**Study:** Continue<br>**Hang-up:** Route to end of script<br>**Wrong Number:** Route to end of script<br>**Other:** Route to end of script
Getting Started	Misc. Content: Getting started: Calling for yourself Question<br><br>Misc. Content: Getting Started calling for yourself option1<br><br>Misc. Content: Getting Started calling for yourself option2	Are you calling for yourself?<br><br>**Yes**<br><br>**No**	Misc Content: Default: Enable Call for Self: Enable /checked Default settings<br><br>**Yes:** Continue<br><br>**No:** Relationship Reveal<br><br>**Settings:**<br>Enable Calling for Self | Caregiver: Checked<br>Calling for Self: Selected

			Calling for Self Position > Before Intro Details: Selected
	Reveal Question (May I get your relationship with the person you are calling for?)	May I get your relationship to the person you are calling for?<br>**Self**<br>**Parent/ Legally acceptable representative**<br>**Friend/Other**	Configuration: Check "Calling for self (relationship reveal)"<br>**Self** Continue<br>**Parent/ Legally acceptable representative** Continue<br>**Friend/Other** proceed to "Calling for Other" Conclusion
Introductory Details	Misc. Content: Introductory Details Question<br>Misc. Content: Introductory Details First Name<br>Misc. Content: Introductory Details Last Name<br>Misc. Content: Introductory Details Phone Number	To get started, may I get your name, zip code and a telephone number in case we get disconnected?<br>Caller's First name: ________________<br>Caller's Middle Initial: ________________<br>Caller's Last name: ________________<br>Caller's Phone Number: ________________<br>Zip Code: ________________	Misc. Content: Custom<br><br>Introductory Details First: Caller First Name<br>Introductory Details Middle Initial: Caller Middle Initial<br>Introductory Details Last Name: Caller Last name<br>Introductory Details Phone Number: Caller Phone Number<br>Introductory Details Zip/Postal Code: Caller Zip/ Postal Code<br><br>Select appropriate choice based on lookup<br>- "No records found" then → New referral prospect (not in database) Continue<br>Previous Caller (found in database) Route to BLOCK 06
Study Overview	Block 2<br>(Study Overview)	We are currently screening participants, between the ages of 35 and 75, that have limited movement in their arms and/or legs up to 6-24 months after having an ischemic stroke.<br><br>PISCES III is a clinical research study to determine whether stem cells injected into a damaged area of the brain can improve function in people with ongoing disability following an ischemic stroke (a stroke that occurs when blood flow to the brain is blocked, such as by a clot). This study is open to U.S. and U.S. territory participants only.<br><br>If you qualify for the study, your participation in the study is a little over one year. This includes up to 11 visits to the study center.<br><br>I will ask you a series of confidential questions about your [your relative or friend's] personal health and present condition and enter your responses into our computer system. You do not have to answer any question you don't want to answer and may end the call at any time. However, your responses will determine if you meet the initial criteria for the study. The questions will take approximately 10 minutes to complete.	

		We respect your privacy. If you decide to provide your answers and contact information during this call, your information will be used only in connection with this research study. If you pre-qualify during this pre-screen, you will have the option to share your information with the PISCES study team for further screening. If you do not qualify during this pre-screen, you will have the option to save your information so we may contact you if the criteria for this study changes in the future.<br><br>The data you provide will be digitally stored on behalf of the research project in the United States in a HIPAA compliant data center and/or on a highly secured server at Duke University Health System in the United States. Data relating to your pre-screening answers will be shared with the sponsor of this study (ReNeuron), who is located in the UK. Your information may be shared with patientprimary, a patient support service that will facilitate (or handle) all planning and payment for your and a caregiver's travel and related expenses to/from site, if you qualify for the study. You may modify or have the data removed at any time by calling us back at [call center callback number] and we can assist you. You can also view our privacy policy any time at http://clinicallrn.com/privacy-policy.html.<br><br>If you willing to answer at this time, I will enter your responses into our computer system.<br><br>Your participation in this study, if eligible, is entirely voluntary.	
Getting Started – Permission to Continue	Misc. Content: Getting Started Question<br>Misc. Content: Getting Started option1<br>Misc. Content: Getting Started option2	Do I have your permission to continue?<br>**Yes**<br>**No**	**Yes:** Continue<br>**No:** Route to "Info Only" Conclusion
Getting Started – Pre-Screening Questions Intro	Misc. Content: Getting Started Patient	To Participant: Now I am going to ask a number of specific questions about you and your health to determine if you may be able eligible to participate in the PISCES III research study.<br><br>To Relative/Caregiver/Friend: Now I am going to ask a number of specific questions about the relative or friend's health to determine if he or she might be eligible to participate in the PISCES III research study. Please note, I will be asking a few questions about your relative or friend's current ability to move their body. If you aren't sure about their ability, or they are not nearby to demonstrate, please keep our number and call us back when you know or they are nearby.	
Pre-Screening Questions	Getting Started Age<br><br>Question Label: Date of Birth	May I have your [your relative or friend's] date of birth?<br><br>**MM/DD/YYYY**	Enable Age | Birthdate: Checked<br>Birthdate: Selected<br>Age Min: 35<br>**If <35:** Route to "DQ Age" Conclusion<br>Age Max: 75<br>**If >75:** Route to "DQ Age" Conclusion

	Block 3(d): Question Label: Source	1. How did you hear about the PISCES III study? (Choose one)<br><br>**A current PISCES III study clinic**<br>**Another doctor or rehabilitation specialist or institution**<br>**Facebook or other social media**<br>**Google or other search engine**<br>**Institutional Website**<br>**Other**	Informational only<br><br>**A current PISCES III study clinic**<br>**Another doctor or rehabilitation specialist or institution**<br>**Facebook or other social media:** Skip to 3<br>**Google or other search engine:** Skip to 3<br>**Institutional Website:** Skip to 3<br>**Other**
	Block 3(d): Question Label: Source Detail	2.<br><br>Agent Note - for a current PISCES III study clinic: Please provide the Study Center or Site ID as provided by the study center (it is a 2 digit number):<br><br>Agent Note - for doctor or rehab specialist: Please provide your doctor or rehab specialist's name, along with their location if possible. For example: Dr. John Smith, Duke Medical Center<br><br>Agent Note - for Other: Can you please provide any details about where you heard about the PISCES III study?<br><br>[open text]	Informational only, open text
	Block 3(d): Question Label: US Resident	3. Do you [Does your relative or friend's] currently reside in the United States?<br><br>**Yes**<br>**No**	**Yes**<br>**No:** Route to "DQ (Alt)" Conclusion
	Block 3(d): Question Label: Most recent stroke (months)	4. How many months ago was your [your relative or friend's] most recent stroke?<br><br>**Less than 3 months ago: DQ**<br>**Between 3 and 6 months ago**<br>**Between 6 and 12 months ago**<br>**Between 12 and 23 months ago**<br>**More than 23 months ago: DQ**	**Less than 3 months ago:** Route to "DQ (Alt)" Conclusion<br>**Between 3 and 6 months ago**<br>**Between 6 and 12 months ago**<br>**Between 12 and 23 months ago**<br>**More than 23 months ago:** Route to "DQ (Alt)" Conclusion
	Block 3(d): Question Label: Most recent stroke (date)	5. What was the date of your [your relative or friend's] most recent stroke? (if unknown, please provide best estimate).<br>Agent note: Try to collect date in Month/Day/Year format. If caller cannot recall at all please type "Unsure"	Informational only, open text
	Block 3(d): Question Label: # of strokes	6. How many stroke(s) have you [has your relative or friend] had?	

		**1**<br>**2-3**<br>**4 or more**	**1**<br>**2-3**<br>**4 or more**
	Block 3(d): Question Label:<br>Type of stroke (recent)	7. What type of stroke was your [your relative or friend's] most recent stroke?<br><br>**Ischemic (blockage of blood flow to the brain, such as from a blood clot)**<br>**Hemorrhagic (bleeding into the brain): DQ**<br>**Not Sure**	**Ischemic (blockage of blood flow to the brain, such as from a blood clot)**<br>**Hemorrhagic (bleeding into the brain) :** Route to "DQ (Alt)" Conclusion<br>**Not Sure**
	Block 3(d): Question Label:<br>Cause of stroke(s)	8. Do you know the cause for your [your relative or friend's] stroke(s)? (Agent Note: Select all that apply – if Not sure only check Not sure)<br>□ **Blockage of blood flow in neck**<br>□ **Related to procedure**<br>□ **Related to heart attack**<br>□ **Atrial fibrillation**<br>□ **Blood clot from leg or other body region**<br>□ **Blood clot from heart**<br>□ **Diabetes**<br>□ **Hypertension**<br>□ **Not sure**	Informational
	Block 3(d): Question Label:<br>Continue to have physical effects due to stroke	9. Do you continue to have physical effects/deficits due to your [your relative or friend's] stroke? (Agent Note: read off the options to the caller and select the one that best describes them)<br>**I have no deficits.: DQ**<br>**I have mild deficits, but am able to do all activities and care for myself.**<br>**I have modest deficits that prevent me from doing all activities of daily living. For example, I need help with bathing or dressing, but I am able to walk without help; I am able to walk with assistance (i.e. another person and/or an assistive device)**<br>**I have significant disability. For example, I cannot walk without help; I am not able to get out of bed.**	**No deficit:** Route to "DQ (Alt)" Conclusion<br>**Mild**<br>**Modest**<br>**Significant**
	Block 3(d): Question Label:<br>Able to shrug shoulders	10. Sometimes symptoms/deficits due to stroke are on one side of your [your relative or friend's] body more than the other. Thinking about the side of your body with the most symptoms/deficits:<br><br>Can you shrug your [Can your relative or friend shrug his/her] affected shoulder?<br>**Yes**<br>**No: DQ**	**Yes**<br>**No:** Route to "DQ (Alt)" Conclusion

	Block 3(d): Question Label:<br>Palm down, able to lift fingers	11. With your [his/her] affected palm down on a table, can you [your relative or friend] start to lift your [his/her] fingers?<br><br>**Yes**<br>**No**	**Yes**<br>**No**
	Block 3(d): Question Label<br>Able to make stroke affected wrist move	12. To Participant: Hold your stroke affected hand with your unaffected hand and try to make your stroke affected wrist move. Can you feel or see it move?<br><br>To Relative/Caregiver/Friend: Have your relative/caregiver/friend hold his/her stroke affected hand with his/her unaffected hand and try to make his/her stroke affected wrist move. Can he/she feel or see it move?<br><br>**Yes**<br>**No**	**Yes**<br>**No**<br><br>Custom DQ Programming for combination of Q11 (No) + Q12 (No) = DQ
Pre-Screening Questions Conclusion	Block 3(d): Question Label<br>Conclusion	13. That concludes the screening questions. Thank you.<br>[Agent note: Click to continue]	
Study Center Locator Intro	Site Locator 1<br>(Getting Started2)	Intentionally Left Blank – Not Applicable<br><br>**Continue**	Intentionally Left Blank – Not Applicable
Study Center Locations Found	Misc. Content: Getting Started3	[Agent note: Select default site and click Continue to proceed]<br>( ) SELECT THIS SITE AND CONTINUE	
Study Center Location Convenient	Misc. Content: Getting Started PCC<br><br>Misc. Content: Getting Clinics option1<br>Misc. Content: Getting Clinics option2	[Agent note: Click Continue and proceed]<br><br>**Continue**	**Continue**: Route to Pre-Qualified Intro & Permission to Continue
No Study Center Locations Found	Misc. Content: Alert Site Search	Intentionally Left Blank – Not Applicable	Intentionally Left Blank – Not Applicable
Alternative Study Center Locator	Misc. Content: Alert Site Search Question<br>Misc. Content: Alert Site Search Opt1<br>Misc. Content: Alert Site Search Opt2<br><br>Misc. Content: Alternate Site Search[zipcode]	Intentionally Left Blank – Not Applicable	Intentionally Left Blank – Not Applicable

Pre-Qualified Intro & Permission to Continue	Misc. Content: PQ Conclusion Intro<br><br>Misc. Content: User Permissions option1<br>Misc. Content: User Permissions option2	Thank you for completing this pre-screening for the PISCES III stroke research study.<br><br>Your answers to the questions indicate that you [your relative or friend] may be eligible for further screening. A member of the PISCES III team will be giving you further study details. Participation is completely voluntary, and you [your relative or friend] can withdraw at any time.<br><br>Are you [Is your relative or friend] interested in further evaluation by the PISCES III team?<br>**Yes**<br>**No**	**Yes:** Continue<br>**No:** Route to No Interest/Info Only Conclusion
Pre-Qualified Permission to Send Info to Study Center	Misc. Content: Permission to Send Info to Site<br><br>Misc. Content: User Permissions2 option1<br>Misc. Content: User Permissions2 option2	Would you like to provide your name and contact information so that your [your relative or friend] information, as well as the answers to the pre-screening questions can be shared with the PISCES III team?<br><br>As a reminder, the information will only be used for the purpose of the study and will not be shared with anyone except study personnel unless you give your permission or except as required by law. If you do not wish to share your information, you will not be considered for further screening for the study.<br>**Yes**<br>**No**	**Yes**<br>**No:** Route to No Interest/Info Only Conclusion

Pre-Qualified User Record	Misc. Content: User Record<br>Misc. Content: User First Name<br>Misc. Content: User Middle Initial<br>Misc. Content: User Last Name<br>Misc. Content: User Address1<br>Misc. Content: User Address2<br>Misc. Content: User City<br>Misc. Content: User State/Province<br>Misc. Content: User Zip/Postal Code<br>Misc. Content: User Primary Phone<br>Misc. Content: User Cell Phone<br>Misc. Content: User Work Phone<br>Misc. Content: User Email<br><br>Misc. Content: User Site<br>Misc. Content: User Site Address	Ok, then let me get some additional information from you and verify your contact information.<br>Please note, to help aid the release of your medical records to ensure we can assess whether you qualify for the study, if you provide your email with the contact information, we will be emailing you a medical release form. Please complete the form by following the instructions in the email. If you require assistance, the PISCES III study team will be able to help.<br><br>**Caller First Name**<br>**Caller Middle Initial**<br>**Caller Last Name**<br>**Caller Address 1**<br>**Caller Address 2**<br>**Caller City**<br>**Caller State**<br>**Caller Zip Code**<br>**Caller Home Phone**<br>**Caller Cell Phone**<br>**Caller Work Phone**<br>**Caller Email**<br>**Best Time to Call**<br>**Alternative Contact Name**<br>**Research Site**<br>**Research Site Address**	Form Fields > Contact Form (User Records)<br>**Caller First Name:** Visible, Required<br>**Caller Middle Initial:** Visible, Not Required<br>**Caller Last Name:** Visible, Required<br>**Caller Phone:** Visible, Required<br>**Caller Zip Code:** Visible, Required<br>**Caller Email:** Visible, Not Required<br>**Caller Address 1:** Visible, Required<br>**Caller Address 2:** Visible, N/A<br>**Caller Cell Phone:** Visible, N/A<br>**Caller Work Phone:** Visible, N/A<br>**Best Time to Call:** Visible, Not Required<br>**Alternative Contact Name:** Visible, Not Required<br>**Doctor:** Not Visible, N/A<br>**Privacy:** Visible, N/A<br>**Opt-In Email:** Not Visible, N/A<br>**Opt-In SMS:** Not Visible, N/A
Pre-Qualified Privacy Options	Misc. Content: User Record Privacy<br><br>Misc. Content: User Record Privacy option1<br>Misc. Content: User Record Privacy option2<br>Misc. Content: User Record Privacy option3<br>Misc. Content: User Record Privacy option4	Your privacy is very important to us. If we are unable to reach you in the future may we leave a message?<br>**Yes**<br>**No**<br>**Voicemail Only**<br>**Person Only**	 <br>**Yes:** Route to Pre-Qualified Permission to Send Info to Study Center<br>**No:** Route to Pre-Qualified Permission to Send Info to Study Center<br>**Voicemail Only:** Route to Pre-Qualified Permission to Send Info to Study Center<br>**Person Only:** Route to Pre-Qualified Permission to Send Info to Study Center
Pre-Qualified Final Conclusion	Conclusions: Prequal Not Sched A	Your name, contact information, and answers to the questions will be forwarded to the PISCES III team. Someone from the PISCES III team will follow-up with you within 2 to 3 business days to provide you with additional information. Please don't forget to check your email for the medical release form.<br><br>If you have not heard from the PISCES III team within 4 business days please call us back at [call center callback number].<br><br>Thank you for calling and have a nice day.	**PCC Notes Field:** multi-line free text<br>**Patient Result Field:** PQ

No Interest/Info Only Conclusion	Conclusions (No Interest): Info Only	Thank you for your interest in learning more about the PISCES III stroke research study. If we can be of further assistance, please contact us at [call center callback number].	**PCC Notes Field:** multi-line free text<br>**Patient Result Field:** PQ No Interest
No Study Center Conclusion Intro	Conclusions (No Site): No Site	Intentionally Left Blank – Not Applicable	Intentionally Left Blank – Not Applicable
No Study Center User Record	Misc. Content: User Record<br>Misc. Content: User First Name<br>Misc. Content: User Middle Initial<br>Misc. Content: User Last Name<br>Misc. Content: User Address1<br>Misc. Content: User Address2<br>Misc. Content: User City<br>Misc. Content: User State/Province<br>Misc. Content: User Zip/Postal Code<br>Misc. Content: User Primary Phone<br>Misc. Content: User Cell Phone<br>Misc. Content: User Work Phone<br>Misc. Content: User Email	Intentionally Left Blank – Not Applicable	Intentionally Left Blank – Not Applicable
No Study Center Privacy Options	Misc. Content: User Record Privacy<br><br>Misc. Content: User Record Privacy option1<br>Misc. Content: User Record Privacy option2<br>Misc. Content: User Record Privacy option3<br>Misc. Content: User Record Privacy option4	Intentionally Left Blank – Not Applicable	Intentionally Left Blank – Not Applicable
No Study Center Final Conclusion	Misc. Content: NO SITE Option 1 closing	Intentionally Left Blank – Not Applicable	Intentionally Left Blank – Not Applicable
Disqualified Conclusion (No Disease)	Conclusions: DQ	Intentionally Left Blank – Not Applicable	Intentionally Left Blank – Not Applicable
Disqualified Conclusion (Age)	Conclusions: DQ (Age)	Thank you for taking the time to answer our questions. Unfortunately. you [your relative or friend] must be between the ages of 35 and 75 years old in order to participate in this clinical research study. Thank you for calling.	**PCC Notes Field:** multi-line free text<br>**Patient Result Field:** DQ Age
Disqualified Conclusion (All Other)	Conclusions: DQ (Alt)	Thank you for taking the time to answer our questions. At this time, one or more of your responses does not meet the requirements for this study. (If caller asks why, read: The combination of all your responses is compared against the study requirements to determine eligibility.)<br><br>With your permission, we would like to keep the information you have provided in this telephone interview on file in case the criteria for this study changes.	

		Please note, if the timing of your last stroke was less than 3 months ago and you met the other requirements for the study, we would like to reach out to you in the near future to see if you're still interested in further screening for the study.<br><br>If you say NO, the information you have provided will be destroyed at the end of this call. Do we have your permission to keep this information?<br>**Yes**<br>**No**	**Yes:** Route to Disqualified User Record<br>**No:** Route to Disqualified Closing (Destroy Info)
Disqualified User Record	Misc. Content: User Record<br>Misc. Content: User First Name<br>Misc. Content: User Middle Initial<br>Misc. Content: User Last Name<br>Misc. Content: User Address1<br>Misc. Content: User Address2<br>Misc. Content: User City<br>Misc. Content: User State/Province<br>Misc. Content: User Zip/Postal Code<br>Misc. Content: User Primary Phone<br>Misc. Content: User Cell Phone<br>Misc. Content: User Work Phone<br>Misc. Content: User Email	Ok, then let me get some additional information from you and verify your contact information:<br>**Caller First Name**<br>**Caller Middle Initial**<br>**Caller Last Name**<br>**Caller Address 1**<br>**Caller Address 2**<br>**Caller City**<br>**Caller State**<br>**Caller Zip Code**<br>**Caller Home Phone**<br>**Caller Cell Phone**<br>**Caller Work Phone**<br>**Caller Email**<br>**Best Time to Call**<br>**Alternative Contact Name**	Form Fields > Contact Form (User Records)<br>**Caller First Name:** Visible, Required<br>**Caller Middle Initial:** Visible, Not Required<br>**Caller Last Name:** Visible, Required<br>**Caller Phone:** Visible, Required<br>**Caller Zip Code:** Visible, Required<br>**Caller Email:** Visible, Not Required<br>**Caller Address 1:** Visible, Required<br>**Caller Address 2:** Visible, N/A<br>**Caller Cell Phone:** Visible, N/A<br>**Caller Work Phone:** Visible, N/A<br>**Best Time to Call:** Visible, Not Required<br>**Alternative Contact Name:** Visible, Not Required<br>**Doctor:** Not Visible, N/A<br>**Privacy:** Visible, N/A<br>**Opt-In Email:** Not Visible, N/A<br>**Opt-In SMS:** Not Visible, N/A
Disqualified Privacy Options	Misc. Content: User Record Privacy<br><br>Misc. Content: User Record Privacy option1<br>Misc. Content: User Record Privacy option2<br>Misc. Content: User Record Privacy option3<br>Misc. Content: User Record Privacy option4	Your privacy is very important to us. If we are unable to reach you in the future may we leave a message?<br>**Yes**<br>**No**<br>**Voicemail Only**<br>**Person Only**	**Yes:** Route to Disqualified Closing (Save Info)<br>**No:** Route to Disqualified Closing (Save Info)<br>**Voicemail Only:** Route to Disqualified Closing (Save Info)<br>**Person Only:** Route to Disqualified Closing (Save Info)
Disqualified Closing (Save Info)	Misc. Content: DQ-ALT Option 1 closing	Your contact information will be used only to contact you regarding this or similar studies.<br>Thank you for calling.	**PCC Notes Field:** multi-line free text<br>**Patient Result Field:** DQ
Disqualified Closing (Destroy Info)	Misc. Content: DQ-ALT Option 2 closing	We will be sure that the information gathered on this call will be destroyed. Thank you for your interest in learning more about the study. If we can be of further assistance, please contact us at [call center callback number].	**PCC Notes Field:** multi-line free text<br>**Patient Result Field:** DQ – Opt. Out
Info Only Conclusion	Conclusions (Info Only): Info Only	Thank you for your interest in learning more about the study. If we can be of further assistance, please contact us at [call center callback number].	**PCC Notes Field:** multi-line free text<br>**Patient Result Field:** Info Only

Calling for Other Conclusion	Conclusions (Calling for Other): Calling for Other	It is important that we speak directly with the person who is interested in the study or the caregiver, friend, or relative who can answer questions about the participant's health condition. Please have them call us back at [call center callback number].<br><br>Thank you for calling.	**PCC Notes Field:** multi-line free text<br>**Patient Result Field:** Calling for Other
Previous PQ Record, No Follow-Up Intro	Misc. Content: Help Lookup Search Text<br>Misc. Content: Info Only1 Question	It appears that you called previously on [DATE] [TIME] and you were [RESULT]. How may I help you?<br>**No Follow-Up from Site**<br>**Other**	 **Patient Result Field:** PQ<br>**No Follow-Up from Site:** Route to Previous PQ Record, No Follow-Up Question<br>**Other:** Route to Info Only Question
Previous PQ Record, No Follow-Up Question	Misc. Content: No Follow-Up Question 1<br>Misc. Content: No Follow-Up Conclusion Option1<br>Misc. Content: No Follow-Up Conclusion Option2	Has a member of the study team called you?<br>**Yes**<br>**No**	 **Yes:** Route to Info Only Question<br>**No:** Route to Previous PQ Record, No Follow-Up – Verify/Update User Record
Info Only Question	Misc. Content: Info Only1 Question	How may I assist you?<br>[Agent: Make notes on caller issue]<br>Thank you for calling.	**PCC Notes Field:** multi-line free text<br>**Patient Result Field:** PQ
Previous PQ Record, No Follow-Up – Verify/Update User Record	Misc. Content: User Record<br>Misc. Content: User First Name<br>Misc. Content: User Middle Initial<br>Misc. Content: User Last Name<br>Misc. Content: User Address1<br>Misc. Content: User Address2<br>Misc. Content: User City<br>Misc. Content: User State/Province<br>Misc. Content: User Zip/Postal Code<br>Misc. Content: User Primary Phone<br>Misc. Content: User Cell Phone<br>Misc. Content: User Work Phone<br>Misc. Content: User Email	Ok, then let me get some additional information from you and verify your contact information:<br>**Caller First Name**<br>**Caller Middle Initial**<br>**Caller Last Name**<br>**Caller Address 1**<br>**Caller Address 2**<br>**Caller City**<br>**Caller State**<br>**Caller Zip Code**<br>**Caller Home Phone**<br>**Caller Cell Phone**<br>**Caller Work Phone**<br>**Caller Email**<br>**Best Time to Call**<br>**Alternate Contact Name**	Form Fields > Contact Form (User Records)<br>**Caller First Name:** Visible, Required<br>**Caller Middle Initial:** Visible, Not Required<br>**Caller Last Name:** Visible, Required<br>**Caller Phone:** Visible, Required<br>**Caller Zip Code:** Visible, Required<br>**Caller Email:** Visible, Not Required<br>**Caller Address 1:** Visible, Required<br>**Caller Address 2:** Visible, N/A<br>**Caller Cell Phone:** Visible, N/A<br>**Caller Work Phone:** Visible, N/A<br>**Caller Best Time to Call:** Visible, Not Required<br>**Alternate Contact Namer:** Visible, Not Required<br>**Doctor:** Not Visible, N/A<br>**Privacy:** Visible, N/A<br>**Opt-In Email:** Not Visible, N/A<br>**Opt-In SMS:** Not Visible, N/A
Previous PQ Record, No Follow-Up – Verify/Update Privacy Permissions	Misc. Content: User Record Privacy<br><br>Misc. Content: User Record Privacy option1<br>Misc. Content: User Record Privacy option2	Your privacy is very important to us. If we are unable to reach you in the future may we leave a message?<br>**Yes**<br>**No**<br>**Voicemail Only**<br>**Person Only**	 <br>**Yes:** Route to Previous PQ Record, No Follow-Up – Conclusion<br>**No:** Route to Previous PQ Record, No Follow-Up – Conclusion

	Misc. Content: User Record Privacy option3<br>Misc. Content: User Record Privacy option4		**Voicemail Only:** Route to Previous PQ Record, No Follow-Up – Conclusion<br>**Person Only:** Route to Previous PQ Record, No Follow-Up – Conclusion
Previous PQ Record, No Follow-Up – Conclusion	Conclusions (No Follow-Up)	We will send a reminder to the PISCES III team with the contact information you just provided. Please contact us again, if you do not hear from the center within 3 business days. Thank you for calling.	**PCC Notes Field:** multi-line free text<br>**Previous Caller Result Field:** PQ<br>**Patient Record Field:** No Follow-Up
Previous Info Only Record	Misc. Content: Help Lookup Search Text	It appears that you called previously on [DATE] [TIME] and you were [RESULT]. How may I help you?<br>**Continue Questionnaire**<br>**Other**	**Previous Caller Result Field:** Info Only<br>**Patient Record Field:** Info Only<br>**Continue Questionnaire:** Route to Getting Started – Permission to Continue<br>**Other:** Route to Info Only Question
Previous No Site Record	Misc. Content: Help Lookup Search Text	It appears that you called previously on [DATE] [TIME] and you were [RESULT]. How may I help you?<br>**Search for Study Center**<br>**Other**	**Previous Caller Result Field:** No Site<br>**Patient Record Field:** No Site<br>**Search for Study Center:** Route to Study Center Locator Intro<br>**Other:** Route to Info Only Question
Previous DQ Record	Misc. Content: Help Lookup Search Text	It appears that you called previously on [DATE] [TIME] and you were [RESULT]. How may I help you?<br>**FAQs**<br>**Other**	**Previous Caller Result Field:** DQ or DQ AGE<br>**Patient Record Field:** DQ or DQ AGE<br>**FAQs:** Route to FAQs<br>**Other:** Route to Info Only Question<br><br>Note: If patient has called previously and disqualified they will not be allowed to go through the screening again<br><br>Help List:<br>1- If no records are displayed, select New Referral and continue.<br>2- If a record is displayed, select this record, select the appropriate choice and continue.<br>3- If more than one record is displayed, discuss with caller to determine which is correct. Select this record, select the proper choice and continue.
Previous PQ No Interest Record	Misc. Content: Help Lookup Search Text	It appears that you called previously on [DATE] [TIME] and you were [RESULT]. How may I help you?<br>**Interested in Study Center Evaluation**<br>**Other**	**Previous Caller Result Field:** No Interest<br>**Patient Record Field:** PQ No Interest<br>**Interested in Study Center Evaluation:** Route to Pre-Qualified User Record<br>**Other:** Route to Info Only Question
Previous PQ Record	Misc. Content: Help Lookup Search Text	It appears that you called previously on [DATE] [TIME] and you were [RESULT]. How may I help you?	**PCC Notes Field:** multi-line free text<br>**Patient Result Field:** PQ

		**FAQs**<br>**Other**	**FAQs:** Route to FAQs<br>**Other:** Route to Info Only Question

FAQ Section:

Response to all STUDY-RELATED questions not included in the screening script or answered in the table below
Answer: That information is not available to us. If you pre-qualify during this phone screening, the study doctor will be able to answer your questions during your first appointment.

If at any point during the call, the caller expresses interest in suicide, or harming themselves/others in any way please advise the caller to hang up and dial 911.

PISCES III Specific FAQs - General	
**FAQ Question**	**FAQ Answer**
I only want information. Can you tell me what the study is about?	PISCES III is a clinical research study to determine if a study drug, which is made from stem cells, will help improve function in people who have limited movement in their arms and/or legs 6–24 months after having an ischemic stroke. Ischemic stroke is the most common type of stroke and occurs when a blood vessel, which supplies blood to the brain is blocked, such as by a clot.<br><br>The drug being studied is called CTX0E03 DP. The study drug is made of stem cells and is injected directly into the brain during surgery. The study will assess the effectiveness of CTX0E03 DP to change the degree of dependency and disability of study participants from baseline, as well as measure a number of safety parameters. CTX0E03 DP has been studied previously in 34 people who have had an ischemic stroke.<br><br>Patients who meet all of the eligibility criteria and enroll will be placed randomly in one of two groups: One will receive the stem-cell injection into the brain during a surgical procedure; the other group will not receive stem cells during a surgical procedure. You will learn which group you are assigned to once the study has been completed.<br><br>All participants will be provided and expected to complete a 12-week physical therapy program and monitored for 12 months. The sponsor will also be conducting a second study which you will be asked to participate in to follow your long-term progress.
What is being tested in this study?	The study drug, CTX0E03 DP, is made from stem cells and is injected in the brain during surgery. The study drug and the equipment being used to inject the stem cells are experimental, meaning they have not been approved for use by the U.S. Food and Drug Administration (FDA). They are being studied in PISCES III as a possible treatment to improve movement in arms and legs after a stroke. The study drug has been studied previously in 34 people who have had an ischemic stroke.
What are stem cells?	Stem cells are cells that can develop into specialized cells or can split to produce more stem cells. In many tissues they act as an internal repair system. The study drug stem cells were made from a single sample of fetal brain tissue that was voluntarily donated to research following a legal termination of pregnancy. These fetal stem cells were genetically modified so that large amounts of stem cells could be produced and tested as a potential therapy for stroke.
How many people will take part?	Approximately 130 people will be involved in this study in the United States at approximately 30 centers.
How do I know if I'm eligible?	You will first need to complete a questionnaire that will ask you details about your stroke, including any remaining disabilities you may have. If you are eligible based on the answers you provide, the Duke Clinical Research Institute will complete a review of your medical records (you will need to complete a medical records release form authorizing the DCRI to receive a copy).<br><br>If after the review you are still found to be eligible for the study, an on-site visit will be scheduled at one of the assessment centers. You will need to undergo some tests to ensure you are eligible for the study.
How long will I be in this study?	Your participation in the study will last a little over one year. You can choose to stop taking part in the study at any time without penalty or loss of any benefits to which you are entitled. During the course of the study the sponsor will also be conducting a y second study which you will be asked to participate in to follow your long-term progress.
Why participate in PISCES III?	If you decide to participate, you will be an important part of the research team and have a vital role in advancing medical knowledge and understanding of stroke therapy.
Who is funding this study?	Funding for PISCES III comes from ReNeuron, a leading stem-cell therapy development company based in the United Kingdom. ReNeuron's primary objective is the development of novel stem cell therapies targeting areas of significant unmet or poorly met medical need.

Is it possible that I may be given placebo instead of the study drug?	**<u>Current Protocol Re-Phrasing:</u>**<br>Yes. To help researchers measure the effects of the study drug, some participants may be part of a "control group" that receives a placebo, while others get the actual study drug. Both groups receive study related medical care.<br><br>Neither the study staff (including the study doctor) or the participant will know whether the participant will receive the study drug or placebo. However, you will learn which group you were assigned to once the study has been completed.<br><br>**<u>Future Protocol Re-Phrasing:</u>**<br>Yes. To help researchers measure the effects of the study drug, some participants may be part of a "control group" that receives a placebo, while others get the actual study drug. Both groups receive study related medical care. There is a 2-in-3 chance of you receiving the investigational procedure and a 1-in-3 chance of receiving a placebo procedure.<br><br>Neither the study staff (including the study doctor) or the participant will know whether the participant will receive the study drug or placebo. However, you will learn which group you were assigned to once the study has been completed.
What is a placebo?	**<u>Current Protocol Re-Phrasing:</u>**<br>If you qualify for the study, you will be randomly assigned to either the study medication or a placebo group. If you are assigned the study medication, you will receive the stem-cell injection into the brain during a surgical procedure; if you are assigned to the placebo group, you will only receive a surgical procedure with no injection. Neither you or the study staff will know your assignment.<br><br>**<u>Future Protocol Re-Phrasing:</u>**<br>If you qualify for the study, you will be randomly assigned to either the study medication or a placebo group. If you are assigned the study medication, you will receive the stem-cell injection into the brain during a surgical procedure; if you are assigned to the placebo group, you will only receive a surgical procedure with no injection. Neither you or the study staff will know your assignment. There is a 66% chance of receiving the investigational procedure and a 33% chance of receiving the procedure with no injection.
Are there any side effects, risks, or complications with the study drug?	If you pre-qualify, a member of the study team can give you information about your concerns.
Will I have to discontinue any current medication?	Please do not discontinue any of your current medications until you have been instructed to do so by the study doctor during an office visit. A member of the study team can give you more information about your medications.
Does my regular doctor have to give me permission?	It is a good idea to notify your regular doctor about your participation, however a referral or permission is not required.
What is Ischemic Stroke?	Ischemic stroke occurs when a blood vessel carrying blood to the brain is blocked, such as by a blood clot. This causes blood not to reach the brain. High blood pressure is the most important risk factor for this type of stroke. Ischemic strokes account for about 87% of all strokes.<br><br>Source: https://www.stroke.org/understand-stroke/what-is-stroke/ischemic-stroke/
What is a Hemorrhagic Stroke?	A hemorrhagic stroke is either a brain aneurysm burst or a weakened blood vessel leak. Blood spills into or around the brain and creates swelling and pressure, damaging cells and tissue in the brain. There are two types of hemorrhagic stroke called intracerebal and subarachnoid.<br><br>Source: https://www.stroke.org/understand-stroke/what-is-stroke/hemorrhagic-stroke/
What is Atrial fibrillation?	Atrial fibrillation (also called AFib or AF) is a quivering or irregular heartbeat (arrhythmia) that can lead to blood clots, stroke, heart failure and other heart-related complications.<br><br>Source: https://www.heart.org/en/health-topics/atrial-fibrillation/what-is-atrial-fibrillation-afib-or-af

Who is the Sponsor? Who is ReNeuron?	This study is sponsored by ReNeuron, a leading, clinical-stage cell therapy development company. Their primary objective is the development of novel stem cell therapies targeting areas of significant unmet or poorly met medical need.<br><br>ReNeuron has used its unique stem cell technologies to develop cell-based therapies for significant disease conditions where the cells can be readily administered "off-the-shelf" to any eligible patient without the need for additional immunosuppressive drug treatments.
Who is Duke Clinical Research Institute (DCRI)?	Duke Clinical Research Institute (DCRI) is the center coordinating the study on behalf of ReNeuron. The mission of DCRI is to develop and share knowledge that improves the care of patients around the world through innovative clinical research.
**PISCES III Specific FAQs – At the end of the call**	
Why did you say I was not eligible to take part?<br><br>***Why have I not qualified for the study?***	Based on your answers, the requirements of the study were not met. All of your responses are compared against the study requirements to determine eligibility.<br>**(If caller is persistent about disqualifying (add the following response)**<br>Clinical research studies are designed with specific criteria to properly evaluate those who take part. If you did not pre-qualify in the pre-screening process, there was some response regarding your symptoms or medical history that indicates this particular study is not the best option for you at this time. We encourage you to speak with your doctor for more information.
What happens to the information I provide in pre-screening?	All information is kept confidential and is maintained in a secure location. Your screening information will be shared with the PISCES III team only if you agree to participate. If you don't agree that the information we collected in this telephone interview be retained in our files, the information you have provided will be destroyed at the end of this call.
I qualified for the study and was referred for further pre-screening, but I have not been contacted me yet.	We will contact the PISCES III study team to follow-up with them regarding this situation. We appreciate you taking the time to screen for this very important research study.
Who is going to contact me and how are they related to you and this study?	You may be contacted by any of the following companies:<br>One company is Clinical Trial Media, which is the company that I work for. We are a privately owned independent company assisting the sponsor, ReNeuron, and the research clinics in the recruitment efforts for this study by providing phone pre-screening support. I am working for a call center based out of New York.<br>The second company is Duke Clinical Research Institute's Prescreening Call Center (PCC), which is another company assisting the sponsor, ReNeuron, and the research clinics in the recruitment efforts for this study. The PCC will be helping you request a copy of your medical records to be released so they can continue the pre-screening efforts we have/will start(ed) today. Once they have reviewed your medical records and if you are still eligible for the study, they will also assist you in organizing your study visits at a research clinic.<br>And finally, you may be contacted by the research clinic staff if you pre-qualify and find a convenient clinic location.
**PISCES III Specific FAQs – Expenses**	
How much do I get paid, or what is the compensation for taking part in this study?	All costs related to your travel, meals, and any overnight hotel stays will be paid for you and, if necessary, your caregiver. Assistance with travel may be arranged if you need to travel long distances to get to the assessment center and/or the surgical center.
Will it cost me anything to take part in this study?	You do not have to pay to take part in this study.
Do I have to pay for anything or will my insurance be billed?	There is no charge to you for participating. A member of the study team can provide you with more information about this.
**PISCES III Specific FAQs – Study Visits**	
Who are the study doctors and where are they located?	The study centers are located in multiple states within the United States. If you pre-qualify during this preliminary screening process, a member of the study team will be calling you to discuss more information, including the location of the study centers. The study center will be determined based on several factors such as:<br>patient and study center location, patient preference, and availability of study center to see patients.

What will happen during the initial visit?	Before you start the study, the doctor or study coordinator will explain the study details and ask you to sign a consent form to take part in this research study.
Where are you located/where would I need to go?	The study centers are located in multiple states within the United States. If you pre-qualify during this preliminary screening process, a member of the study team will be calling you to discuss more information, including the location of the study centers. The study center will be determined based on several factors such as: patient and study center location, patient preference, and availability of study center to see patients.
Can I go to my appointments after work or on the weekends?	If you pre-qualify for this study, you can discuss this with the study center.
How long will the appointments last?	The study visits vary in length depending on the type of visit. Several tests will be performed at each visit. If you pre-qualify for this study, you can discuss this with the study center.
What will I have to do at the study visits?	That will vary depending on the visit. Several tests will be performed at each visit.
**Medical Records Specific FAQs**	
Questions about Medical Records	PISCES III: Instructions for Requesting Medical Records<br><br>Thank you for completing the survey.<br><br>In order to continue, we need to collect a copy of certain medical health records so that we can determine if you meet the requirements to be seen at one of our assessment centers.<br><br>Please do the following to start the process of obtaining and providing to us a copy of your medical health records.<br>1. Download and print the instructions, as well as, the Medical Records Release Form<br>2. Complete and sign the form, then send to the PISCESIII Call Center (PCC) by fax at 919-385-7594 or email at PISCES3@duke.edu<br>3. Locate the Medical Records Department of the hospital(s) and/or any medical facilities that diagnosed and treated your stroke. You can do this by either:<br>a. You can call the main information phone number of the hospital/facility and request to be transferred to the Medical Records Department, or<br>b. You can do an internet search for the name of your hospital/facility to determine how to contact them and have your medical records released to us. Many Medical Records<br>c. Departments can be contacted by email.<br>4. Contact the Medical Records Department. Tell them you are interested in participating in a clinical trial and need a copy of your medical records to be sent to the PISCESIII Call Center via fax at 919-385-7594 or email at PISCES3@duke.edu. Many hospitals/facility will accept the Medical Records Release Form we provided to you, but it is possible you will need to use the hospital/facility's own release form. Follow the instructions they provide in order to obtain the needed records. Please contact the PCC if you or the facility need any assistance in completing the medical records release.<br><br>You should request the following records from your time in the hospital/facility due to your stroke:<br>a. Hospital Discharge Summary<br>b. Reports from procedures, surgeries, laboratory tests, biopsies and radiology images<br>c. Clinic notes/visits<br><br>The PISCES IIII Study Team will contact you by phone to follow up on the medical record release and assist if needed. Once they receive the completed request form, and your medical records from the hospital/facility, they will review the information and let you know whether or not you will be asked to visit one of the assessment centers.
	You should have received an email from [email] with the medical record form.
**Non-Study Specific FAQs**	
What is a research study?	A clinical research study is a carefully designed scientific evaluation of a study drug conducted by doctors. Clinical research studies help to answer important medical questions, such as how a new study drug acts in the body, how it affects certain diseases or conditions, and whether or not it is safe for wider use.

What is the benefit in joining a research study?	The goal of clinical trials is to determine if the treatment, prevention, and behavior approaches are safe and potentially effective. People take part in clinical trials for many reasons. People with illnesses or diseases take part to help others in the future, and because they are looking at options to address their disease. Clinical trials may offer hope for many people and a chance to help researchers potentially find better medications for others in the future.
I am calling regarding your advertising; do you need any help with advertising? (Used when the call center receives cold calls from other agencies/reps/etc.)	All advertising needs have been handled, thank you.
What is meant by 'study drug'?	A study drug is one that is used in a clinical research study and may be studied for its safety and potential effectiveness. The study drug and the equipment being used to inject the stem cells are experimental, meaning they have not been approved for use by the U.S. Food and Drug Administration (FDA). They are being studied in PISCES III as a possible treatment to improve movement in arms and legs after a stroke. The study drug has been studied previously in 34 people who have had an ischemic stroke.
Response to requests for medical advice.	Agent Note - If new caller: I am not permitted to offer medical advice. It is best that you discuss this type of question with your doctor or the study doctor during your initial visit.<br><br>Agent Note - If caller states they have already pre-qualified proceed to warm transfer verbiage.
What if I decide I don't want to participate?	Participation is strictly voluntary. You may withdraw at any time.
Who do you work for? / Where do you work?	You are speaking with a Clinical Trial Media representative; we are a privately owned independent company working with the sponsor, ReNeuron, and the research clinics in the recruitment efforts for this study by providing phone pre-screening support. I am working for a call center based out of New York.
Why do you need my email address?	We will use it as another way of contacting you. If you are uncomfortable providing it, then we do not have to include it.
By answering screening questions am I committed to enroll in the study or to complete the study?	No. All research is completely voluntary and you have the right not to participate at any time. You can discuss this with a member of the study team at your first visit to the office.

**Outbound Scripts**	
**Label**	**Text**
Outbound Intro:<br>Returning call from an afterhours/no agents available voicemail	Hello, may I speak with [name]?<br>(Note: confirm you are speaking to the person intended. Once confirmed, proceed. If the person cannot be confirmed then proceed to call termination sequence.)<br>Hello, my name is (Operator Name). I am a nurse (or other applicable credential) calling in response to your voicemail regarding the PISCES III study for people who have had an ischemic stroke. I'm wondering if you would be interested in learning more about the study?<br>If yes: (continue to screener)<br>If no: (choose from dropdown, wrong number, hangup, or other and route to close script)<br>If no answer or not available: (Leave message by saying):<br>Hello, my name is (Operator Name) and you recently left us a voicemail to inquire about clinical research studies. Please call us back at (call center call back number) to receive more information about this study. Any of the agents that answer can assist you. That number again is [call center callback number]. Thank you for your interest and have a nice day.

	(choose from dropdown, left message and route to close script)
Outbound Intro:<br>Previous PQ No Follow-Up Alternate Site Available<br><br>(Note: Agent will have next available site pre-determined/provided to them prior to the outbound call)<br><br>For live answer. VMs will not mention study indication.	Hello, may I speak with [name]?<br>(Note: confirm you are speaking to the person intended. Once confirmed, proceed. If the person cannot be confirmed, then end call.)<br>Hello, my name is [Nurse Name]. I am a nurse (or other applicable credential) calling regarding the Pisces III study for people who have had an ischemic stroke. Our records indicate that you recently pre-qualified for the study. We are calling because the study center your information was previously sent to is [currently at capacity evaluating participants/now closed/no longer accepting patients]. Would you be interested in having your information sent to another study center in your area that is accepting participants?<br><br>**If Yes:** Discuss next closest site option and confirm interest.<br>[Referral confirmation]<br>Your name, contact information, and answers to the questions will be forwarded to the study center's office. Someone from the study center will follow-up with you within a few days to provide you with additional information. If you have not heard from the study center's office within a few days, please call us back at [call center callback number]. Thank you and have a great day. [Agent Note: choose appropriate disposition]<br><br>**If No:** Ok, thank you for your time. Have a great day.
Outbound Intro:<br>Previous PQ No Follow-Up From Site<br>(Note: Agent will have referral info determined/provided to them prior to the outbound call)<br><br>For live answer. VMs will not mention study indication.	Hello, may I speak with [name]?<br>(Note: confirm you are speaking to the person intended. Once confirmed, proceed. If the person cannot be confirmed, then end call.)<br>Hello, my name is [Nurse Name]. I am a nurse (or other applicable credential) calling regarding the Pisces III study for people who have had an ischemic stroke. Our records indicate that you recently pre-qualified for the study. We are calling because due to overwhelming response, the Duke Clinical Research Institute's Prescreening Call Center is experiencing a delay in follow-up. It may be a few more [days/weeks] until the Duke Clinical Research Institute's Prescreening Call Center will be reaching out to you. We thank you for your patience. Have a great day.
Outbound Intro:<br>Previous DQ Stroke less than 3 months<br>(Note: Agent will have referral info determined/provided to them prior to the outbound call)<br><br>For live answer. VMs will not mention study indication.	Hello, may I speak with [name]?<br>(Note: confirm you are speaking to the person intended. Once confirmed, proceed. If the person cannot be confirmed, then end call.)<br>Hello, my name is [Nurse Name]. I am a nurse (or other applicable credential) calling regarding the Pisces III study for people who have had an ischemic stroke. Our records indicate that you previously disqualified for the study due to the timing of your last stroke. We are calling because now that some time has passed, you may be eligible for further screening. Are you still interested in further screening for this study?<br><br>**If Yes:** Continue to pre-screener<br><br>**If No:** Ok, thank you for your time. Have a great day.
Outbound Intro:<br>Study site closed with no other sites available.	Hello! May I speak to [name]?<br>(Note: confirm you are speaking to the person intended. Once confirmed, proceed. If the person cannot be confirmed, then end call.)<br>I am calling regarding the Pisces III study for people who have had an ischemic stroke for which you [recently] screened. We are calling to let you know that the study center [you chose/has been selected for you] has closed enrollment for this study, so you will not be hearing from them. Unfortunately, [we do not have any other study centers in your area/we are unable to re-arrange travel to another study center]. We appreciate you taking the time to screen for this very important research study. Have a nice day/evening.

For live answer. VMs will not mention study indication.	
Outbound Intro:<br>Study closed<br><br>For live answer. VMs will not mention study indication.	Hello! May I speak to [name]?<br>(Note: confirm you are speaking to the person intended. Once confirmed, proceed. If the person cannot be confirmed, then end call.)<br>I am calling regarding the Pisces III study for people who have had an ischemic stroke for which you [recently] screened. We are calling to let you know that the study is now closed to enrollment, so you will not be hearing from the study center. We appreciate you taking the time to screen for this very important research study. Have a nice day/evening.

Warm Transfer Scenarios	
Anything PRIOR to their first visit which are not related to the survey e.g.<br>• pre-screening (of med records)<br>• mRS qualification telephone call<br>• release of medical records<br>• organising their first visit<br>• anything related to travel arrangements.<br>• If prior to their first visit, then they can be referred to the PCC<br>• if caller cannot remember their assessment site's contact information.	This question would be handled by Duke Clinical Research Institute's Prescreening Call Center. I can try to transfer you to them now. If we do not get through, I'll leave a voicemail.<br><br>In just a moment, I will be transferring you, but before I do, please note the following name and number in case you need to contact them on your own:<br>Duke Clinical Research Institute's Prescreening Call Center 833-202-5380.<br><br>Okay, now I will attempt to transfer you. One moment while I transfer you.<br><br>Agent Note: click transfer and insert the number shown above. Stay on call while transferring.<br><br>LIVE If answered: Hello, this is [agent first name] calling on behalf of the PISCES III study. I have a caller on the phone that would like to discuss [insert topic]. Is this a good time?<br>Agent Note: if yes, state caller's name and then say:<br>You may go ahead now. Thank you and have a nice day.<br>Agent Note: click hang-up then 'end call for me'<br><br>If NO ANSWER or BUSY signal click on hang-up then 'end transfer', then tell the caller:<br>The Duke Clinical Research Institute's Prescreening Call Center is unavailable at this time. You can try reaching them at 833-202-5380 at a later time.<br><br>Thank you for calling and have a nice day.<br>[click on hang up]<br><br>If VOICEMAIL:<br>Leave a message by saying:<br>Hello, this is [agent first name] calling on behalf of the PISCES III study. [Caller name] has a question about [topic], can you please call [him/her] back at [contact phone]. [He/she] will expect a call back in the next few days. Thank you.<br>PCC Note: Click on hang-up then 'end transfer' then tell the caller<br>The Duke Clinical Research Institute's Prescreening Call Center is unavailable at this time. I've left them a message with your name and contact information and someone from the call center will contact you within the next few days.<br><br>Thank you for calling and have a nice day.

	PCC Note: click hang-up then ‘end call for me’<br><br>BUSY<br>DECLINED TRANSFER<br>NO ANSWER / ABANDONED<br>SUCCESSFUL<br>VOICEMAIL
