# Supplementary material for: Experience with a hybrid recruitment approach of patient-facing web portal screening and subsequent phone and medical record review for a neurosurgical intervention trial for chronic ischemic stroke disability (PISCES III)
Source: Trials. 2024 Feb 28;25:150. doi: 10.1186/s13063-024-07988-z (PMC10900735; doi:10.1186/s13063-024-07988-z)
Supplement: Supplementary file 3 — Additional file 3. Summary of Analysis of mRS Approaches and Agreement [file 13063_2024_7988_MOESM3_ESM.pdf]

Appendix 3 - Summary of Analysis of mRS Approaches and Agreement

mRS scoring tool historical development Timeline:

Rankin scale introduced 1957[1]

Addition of 0 and 6 categories for use in RCTs 1988[3]

Concerns around interrater and intrarater reliability result in development of structured interview 2002-5[4, 5]

An effort to provide more structure and guidance to the mRS assessment was initiated 2006[2]

Saver modification to make simpler and faster across varied raters developed RFA 2010[6]

Development of the simplified mRS questionnaire (smRSq) 2010[7]

Validation of the smRSq over phone and correlation with QOL 2011[8]

Validation that smRSq correlates with stroke severity (NIHSS) 2013[9]

Postal card (mRS and smRSq) and telephone (RFA) questionnaires vs FtF shows agreement 2012[10]

Validation of RFA in mobile app 2015[11]

Based on the above literature, we used the smRSq to screen mRS by phone at the PCC. This was due to the simplicity of the screening, the more inclusive nature of the questions, validation over the phone, and validation with stroke severity and QOL. However, training materials and RFA forms were used at the sites (through VeraSci) due to availability of training materials and standard forms. Current experience suggests, however, there is **not** good agreement between these screening tools. Specifically, the smRSq was more likely to judge that a patient would be eligible for the study based on a mRS of 3, whereas the site assessment based on the RFA would be more likely to score a 2, thus resulting in disqualification at the initial site visit. This prompted this evaluation of the data and experiences within the PISCES III trial.

Abbreviations:
FtF – face –to-face
mRS – Modified Rankin Score (0-6 scale)
PCC – Primary Chart Screening Center at DCRI for PISCES 3 trial
QOL – Quality of Life
smRSq – Simplified Modified Rankin Score Questionnaire
RFA – Rankin Focused Assessment

Data review:

Inter-rater reliability using the original mRS itself is modest, 43% exact agreement with 50% discordance by greater than one category. In particular, discrimination between mRS of 2 vs 3 is difficult. [4, 5] The early

studies by Wilson and colleagues, showed that for mRS categories 2 and 3 there is particularly high disagreement with 30-50% of 3 being classified as 2 by the a second independent rater. [4, 5] Use of a structured interview improved agreement significantly to 81% agreement overall. [4, 5]

Agreement between the patient reported smRSq and phone RFA is also modest with 65% exact agreement. [10] This data clearly shows the difficulty we are having with our current approach in POISCES III. We are phone screening with the smRSq and then using the RFA tool by phone or at Visit 1. There was enormous variation in self-reported 3s on the smRSq as well as the RFA determined 3s in this prior work (see Table 3 [10]).

Thus, while interrater agreement is high for both the RFA and the smRSq the agreement is less when subjects rate themselves on these tools. The RFA seems to be more affected by patient self-reporting remotely. For example, the agreement between provider raters using RFA and FtF assessment is 94%, however between the self-reported RFA via Mobile App and provider-based FTF is 62.5%.[11] For the smRSq agreement when compared between FtF assessment by different raters was 78% and 82% between phone administered and FTF assessments.[10]

Of interest is the way patients self-report when compared to provider ratings. This can be seen in studies using postal cards, telephone, and mobile apps compared to the RFA or smRSq in person. Patients generally rate themselves as more disabled than the independent rater. For example, in a Dutch study, the structured interview tool for mRS was translated and then used by phone or face-to-face and ratings were compared. More than half of the phone established mRS 3 were classified as mRS 2 at the FtF.[12] Similar results were seen when comparing self-reported mRS using tickboxes on a postcard compared to the rating obtained by using the RFA by phone. Of self-reported mRS 3, 28% were reclassified as 1 or 2, and RFA classified mRS 3 was self-classified as < 3 19% of the time and as 4 25.5% of the time. This is an exact agreement of only 55%.[10] The same design using the smRSq on the postcard showed self-reported 3 was rescreened as < 3 20% of the time. Even the 4s were re-classed as 3s 55.5% of the time, and one case as a 1. [10] This is a reflection of our PISCES III experience in applying the same tools for screening, 16 of 61 subjects (26%) in 3-4 by self-report are less disabled by 1 category on the RFA screen. Mobile apps did not show any improvement in patient and provider agreement. [11] Subjects were given the RFA tool on a mobile phone app and allowed to answer questions on their own. The subjects were then seen in clinic and scored using the same tool within a week. Not surprisingly, 6 of 11 (54.5%) self-reported 3s were re-classed to 1 or 2 at the clinic visit. [11]

How MRS 3 is defined in the standard mRS scale is different compared to the RFA sub questions for mRS 3. This quote is from the original definition with guidance proposed by Wilson and colleagues.[2]

"Moderate disability; requiring some help, but able to walk without assistance – Note: assistance is essential for using public transport to get around, but is not essential for walking, eating, maintaining routine daily hygiene, using the toilet, etc."

Part 3 ASSISTANCE TO LOOK AFTER OWN AFFAIRS	
	Assistance includes physical assistance, or verbal instruction, or supervision by another person.<br>Central issue--Could the patient live alone for 1 week if he/she absolutely had to?
**3.1**	**Is assistance ABSOLUTELY essential for preparing a simple meal?** (For example, able to prepare breakfast or a snack)
**3.2**	**Is assistance ABSOLUTELY essential for basic household chores?** (For example, finding and putting away clothes, clearing up after a meal. Exclude chores that do not need to be done every day, such as using a vacuum cleaner.)
**3.3**	**Is assistance ABSOLUTELY essential for looking after household expenses?**
**3.4**	**Is assistance ABSOLUTELY essential for local travel?**<br>(Patients may drive or use public transport to get around. Ability to use a taxi is sufficient, provided the person can phone for it themselves and instruct the driver.)
**3.5**	**Is assistance ABSOLUTELY essential for local shopping?** (Local shopping: at least able to buy a single item)

Section 3 of RFA tool S2 from Saver et al, 2010[6]. A “yes” on any of the items 3.1-3.5 scores an mRS 3

There is a dramatic difference in the level of function between the mRS 3 in the original scale and the 3 defined in the RFA. The first definition seems to imply that only if assistance is needed for use of public transport are they a 3, and if they occasionally need help with any other activities but it is not essential then they may be a 2. The RFA puts a time limit of 1 week on the dependence and implies that any level of need in activities of daily living scores a 3. Neither help determine the score for those that are wheelchair dependent for longer distance travel, but can walk around their home with hand rails as an example. Individual physician interpretations are not consistent between raters which further worsens agreement between these rating tools.

The take home message from this review of data and the literature is that patients may view themselves as more disabled than providers do. The use of the RFA tool drives patients into the less impaired (≤3) categories because of the wording and providers are more likely to adhere to the more rigid interpretations of the RFA mRS 3 questions than patients. This results in higher disagreement between patient and provider responses to the RFA questions and a specific bias towards relative under-reporting of symptom severity by third party assessors at the site (using the Saver RFA) relative to assessment by phone or by personal survey using the smRSq. Further, the smRSq is more consistent with patient self-report of level of disability. It is also clear that it is not just a function of the tool used, but differences in the understanding of what disability means and how it impacts daily life. Better alignment of definitions of disability between patients and providers is required to improve the agreement, regardless of the tool used to rate it.

Conclusions and Recommendations for Changes in PISCES III Screening:

Based on this review and multiple phone calls with current trial sites to discuss this issue it would seem the issue of agreement on mRS score is multifactorial. The disagreement, which is currently running at 38.5%, is due to variation in patient responses to questions, use of different screening tools by PCC and sites, and different expectations for the target participant mRS categories by the patients, site investigators and the screening teams.

To begin to control this, we took the following actions:

1. Clearly define the target population for the trial through a clear definition of the mRS categories of 3 and 4. Then share this with the site teams and provide training around these definitions.
2. All screening should be done using the same screening tools at site-based screening and central screening. We implemented using the smRSq as it is more inclusive and consistent with patient self-reported disability perceptions, while still identifying disabled (mRS 3) participants that are clearly definable and separate from mRS 2. These scores have been correlated with QOL and stroke severity as well and are in good alignment to the proposed categorical definitions.
3. Set appropriate expectations with the site investigators, there is documented agreement between phone smRSq scores and FTF smRSq scores of 82%, though it was worse among faculty than among residents. Thus, it is critical that site PIs also agree with the proposed definitions and are willing to include subjects with the level of disability as defined in the proposed categorical and smRSq definitions.

Will use these definitions of the mRS categories:

Developed based on Table 1 Guidance Scheme from Shinohara et al, cerebrovasc dis, 21, 2006 [2]		
mRS	Clinical Description	Guidance Comments
0	No symptoms	No subjective symptoms or objective signs
1	No significant disability despite symptoms; able to carry out all usual duties and activities	Despite subjective symptoms or objective signs, there has been no change in the person's ability to work or perform activities of daily living compared to before the stroke
2	Slight Disability; unable to carry out all previous activities but able to look after own affairs without assistance	Despite some limitations in the person's ability to carry out his/her usual duties and activities compared to before the stroke, he/she can lead an independent life
3	Moderate disability; requiring some help, but able to walk without assistance	Assistance (physical or verbal) or supervision is essential for using public transport to get around, but is not essential for walking on a flat surface (with or without cane/walker), eating, maintaining routine daily hygiene, using toilet, etc.
4	Moderately severe disability; unable to walk on a flat surface without assistance, and unable to attend to own bodily needs without assistance.	Assistance (physical or verbal) or supervision is essential for walking on a flat surface (with or without cane/walker), eating, maintaining routine daily hygiene, using toilet, etc., but constant care is not required
5	Severe disability; bedridden, incontinent and requiring constant nursing care and attention	Assistance is necessary at all times

Will use this smRSq screening tool[7] with the one modification suggested by the conference call review with site investigators of adding a time duration to the first question of “one week”.

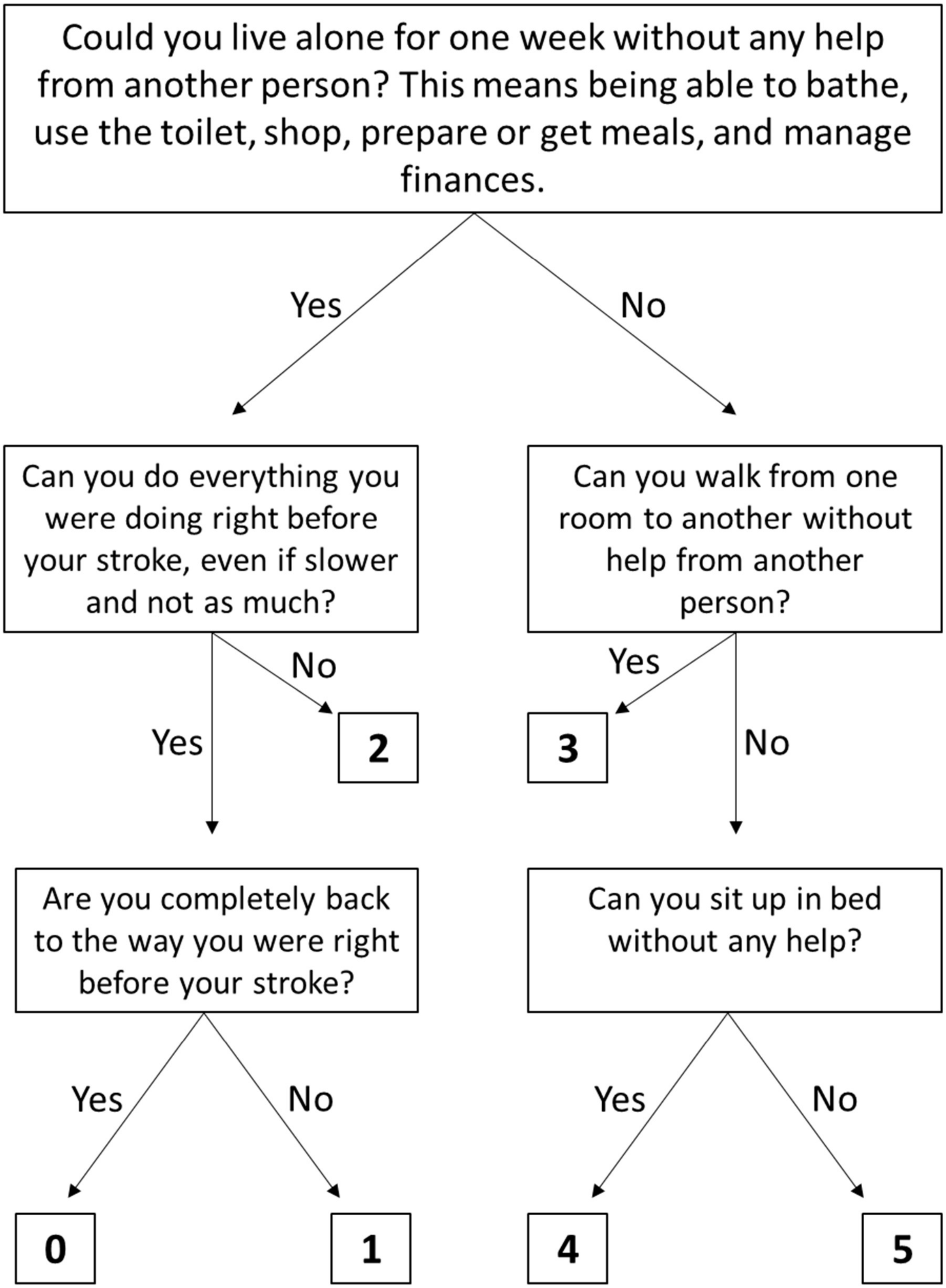

References

1. Rankin J. Cerebral vascular accidents in patients over the age of 60. Ii. Prognosis. *Scott Med J*. 1957;2:200-215
2. Shinohara Y, Minematsu K, Amano T, Ohashi Y. Modified rankin scale with expanded guidance scheme and interview questionnaire: Interrater agreement and reproducibility of assessment. *Cerebrovasc Dis*. 2006;21:271-278
3. van Swieten JC, Koudstaal PJ, Visser MC, Schouten HJ, van Gijn J. Interobserver agreement for the assessment of handicap in stroke patients. *Stroke*. 1988;19:604-607
4. Wilson JT, Hareendran A, Grant M, Baird T, Schulz UG, Muir KW, et al. Improving the assessment of outcomes in stroke: Use of a structured interview to assign grades on the modified rankin scale. *Stroke*. 2002;33:2243-2246
5. Wilson JT, Hareendran A, Hendry A, Potter J, Bone I, Muir KW. Reliability of the modified rankin scale across multiple raters: Benefits of a structured interview. *Stroke*. 2005;36:777-781
6. Saver JL, Filip B, Hamilton S, Yanes A, Craig S, Cho M, et al. Improving the reliability of stroke disability grading in clinical trials and clinical practice: The rankin focused assessment (rfa). *Stroke*. 2010;41:992-995
7. Bruno A, Shah N, Lin C, Close B, Hess DC, Davis K, et al. Improving modified rankin scale assessment with a simplified questionnaire. *Stroke*. 2010;41:1048-1050
8. Bruno A, Akinwuntan AE, Lin C, Close B, Davis K, Baute V, et al. Simplified modified rankin scale questionnaire: Reproducibility over the telephone and validation with quality of life. *Stroke*. 2011;42:2276-2279
9. Bruno A, Close B, Switzer JA, Hess DC, Gross H, Nichols FT, 3rd, et al. Simplified modified rankin scale questionnaire correlates with stroke severity. *Clin Rehabil*. 2013;27:724-727
10. Dennis M, Mead G, Doubal F, Graham C. Determining the modified rankin score after stroke by postal and telephone questionnaires. *Stroke*. 2012;43:851-853
11. Cooray C, Matusevicius M, Wahlgren N, Ahmed N. Mobile phone-based questionnaire for assessing 3 months modified rankin score after acute stroke: A pilot study. *Circ Cardiovasc Qual Outcomes*. 2015;8:S125-130
12. Janssen PM, Visser NA, Dorhout Mees SM, Klijn CJ, Algra A, Rinkel GJ. Comparison of telephone and face-to-face assessment of the modified rankin scale. *Cerebrovasc Dis*. 2010;29:137-139
